# Phylogenetic and Selection Analysis of an Expanded Family of Putatively Pore-Forming Jellyfish Toxins (Cnidaria: Medusozoa)

**DOI:** 10.1093/gbe/evab081

**Published:** 2021-04-23

**Authors:** Anna M L Klompen, Ehsan Kayal, Allen G Collins, Paulyn Cartwright

**Affiliations:** 1 Department of Ecology and Evolutionary Biology, University of Kansas, Lawrence, USA; 2 Department of Invertebrate Zoology, National Museum of Natural History, Smithsonian Institution, Washington, District of Columbia, USA; 3 Sorbonne Université, CNRS, FR2424, Station Biologique de Roscoff, Place Georges Teissier, 29680, Roscoff, France; 4 National Systematics Laboratory of NOAA’s Fisheries Service, Silver Spring, Maryland, USA

**Keywords:** venom, Cnidaria, Medusozoa, jellyfish toxins, pore-forming toxins, transcriptomics

## Abstract

Many jellyfish species are known to cause a painful sting, but box jellyfish (class Cubozoa) are a well-known danger to humans due to exceptionally potent venoms. Cubozoan toxicity has been attributed to the presence and abundance of cnidarian-specific pore-forming toxins called jellyfish toxins (JFTs), which are highly hemolytic and cardiotoxic. However, JFTs have also been found in other cnidarians outside of Cubozoa, and no comprehensive analysis of their phylogenetic distribution has been conducted to date. Here, we present a thorough annotation of JFTs from 147 cnidarian transcriptomes and document 111 novel putative JFTs from over 20 species within Medusozoa. Phylogenetic analyses show that JFTs form two distinct clades, which we call JFT-1 and JFT-2. JFT-1 includes all known potent cubozoan toxins, as well as hydrozoan and scyphozoan representatives, some of which were derived from medically relevant species. JFT-2 contains primarily uncharacterized JFTs. Although our analyses detected broad purifying selection across JFTs, we found that a subset of cubozoan JFT-1 sequences are influenced by gene-wide episodic positive selection compared with homologous toxins from other taxonomic groups. This suggests that duplication followed by neofunctionalization or subfunctionalization as a potential mechanism for the highly potent venom in cubozoans. Additionally, published RNA-seq data from several medusozoan species indicate that JFTs are differentially expressed, spatially and temporally, between functionally distinct tissues. Overall, our findings suggest a complex evolutionary history of JFTs involving duplication and selection that may have led to functional diversification, including variability in toxin potency and specificity.

Significance

Medically relevant jellyfish, primarily cubozoans, are known to possess highly hemolytic and cardiotoxic pore-forming toxins called jellyfish toxins, but the diversity of these toxins and the extent of their distribution across Cnidaria is unclear. Our analyses of publicly available transcriptomes show that these toxins are widely distributed across the cnidarian subphylum Medusozoa, with rampant duplication followed by episodic gene-wide positive selection within the highly toxic Cubozoa. These findings provide a framework to better understand the evolutionary history of jellyfish toxins across Medusozoa, and specifically highlight how these widespread medusozoan toxins may have evolved to be particularly dominant and potent in cubozoan venoms.

## Introduction

**Fig. 1.**
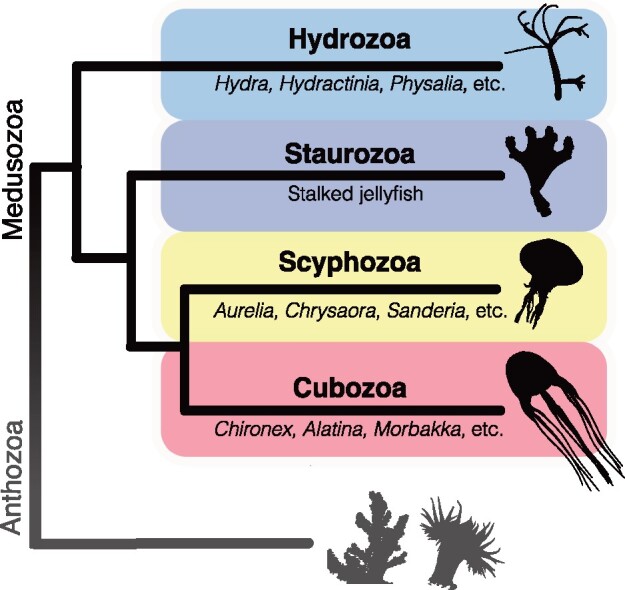
Simplified phylogenetic tree of Cnidaria. Based on Zapata et al. (2015) and Kayal et al. (2018), excluding the Endocnidozoa.

Cnidarians possess a remarkable ability to catch and subdue prey due to a complex cocktail of toxins known as venom, which is concentrated in a unique delivery apparatus called a nematocyst housed within cells called nematocytes. Among the three major cnidarian clades, anthozoans (anemones, stony corals, octocorals, etc.) dominantly display low-molecular weight neurotoxins within their venoms, whereas medusozoan (jellyfish, box jellies, and hydroids) venoms contain comparatively more cytolytic and enzymatic proteins (Hessinger 1988; Rachamim et al. 2015), though there is a sampling bias in cnidarian venoms toward anthozoans (Ashwood et al. 2020). The third cnidarian clade, the parasitic Endocnidozoa (Chang et al. 2015; Kayal et al. 2018), does not appear to express any venom (Turk and Kem 2009), though research into this question is still ongoing. Among the most notorious and emblematic cnidarians, in terms of human envenomation, are the jellyfish-bearing species of the clade Medusozoa, which includes the classes Scyphozoa (true jellyfish), Cubozoa (box jellyfish), Staurozoa (stalked jellyfish), and Hydrozoa (hydroids, hydromedusae, and siphonophores) (fig. 1). Human encounters with some medusozoan species can result in painful and sometimes medically dangerous, stings. Many jellyfish species known to be especially harmful to humans belong to the class Cubozoa or box jellyfish (Yanagihara et al. 2016). Envenomation from some box jellyfish species can cause cutaneous pain, tissue necrosis, intense immunological responses, and cardiovascular or respiratory complications severe enough to be fatal (Fenner and Williamson 1996; Brinkman and Burnell 2009; Turk and Kem 2009; Yanagihara et al. 2016).

The harmful effects of cubozoan envenomations have been linked to the presence of a specific family of pore-forming toxins (PFTs), described as the most potent cnidarian toxin families currently known (Jouiaei, Yanagihara, et al. 2015). Broadly, PFTs are cytolytic proteins that disrupt the cell membrane through the creation of pores, typically in three sequential steps as follows: 1) soluble monomeric protein units are synthesized and released by the organism, 2) bind to the lipids of the target membrane cell, where they form a nonfunctional oligomer complex (often called a prepore), and 3) conformational changes in the prepore allow the membrane–bound complex to insert into the cell membrane (Anderluh and Lakey 2008). A cnidarian-specific PFT family called the jellyfish toxins or JFTs (∼40–50 kDa) was first identified in several cubozoan species (reviewed in Nagai 2003): CrTX-A from *Carybdea brevipedalia* Kishinouye, 1891 (Nagai, Takuwa-Kuroda, et al. 2000), CaTX-A from *Alatina alata* Reynaud, 1830 (Nagai, Takuwa-Kuroda, Nakao, et al. 2000), and CqTX-A from *Chironex yamaguchii* (Lewis and Bentlage, 2009; Nagai et al. 2002). Of note, cubozoan species names used throughout this study reflect updated taxonomic understanding (Lewis and Bentlage 2009; Bentlage et al. 2010; Toshino et al. 2015; Lawley et al. 2016). Several toxins within this family were subsequently cloned from the Australian sea wasp *Chironex fleckeri* Southcott, 1956, a cubozoan notoriously dangerous to humans, including CfTX-1, CfTX-2 (Brinkman and Burnell 2007), CfTX-A, and CfTX-B (Brinkman et al. 2014). A total of 15 JFT isoforms were described in the transcriptome for *C. fleckeri*, 13 of which were also identified through proteomic analysis (Brinkman et al. 2015), whereas transcriptomic analyses recovered 11 homologs from the cubozoan *A. alata* (Lewis Ames et al. 2016). A list of previously characterized toxins that were reported as putative JFTs are shown in supplementary table S1, Supplementary Material online.

PFTs can adopt two conformations, both of which are present in bacteria and metazoans (Peraro and van der Goot 2016): a β-barrel pore (β-PFTs) or variable clusters of α-helices that form a barrel structure (α-PFTs). Structural models for JFTs from *C. fleckeri* showed the greatest structural homology to the N-terminal domain of insecticidal 3d-Cry toxins found in the Gram-positive soil-dwelling bacterium *Bacillus thuringiensis*, suggesting a pore-formation mechanism through α-helices (Brinkman and Burnell 2007, 2009). A structural analysis of a partial CfTX-1 peptide (CfTX-1_22-47_) spanning the predicated amphiphilic α-helix region (residues 24-33) did not form an α-helix in isolation, but the authors note that further work on the full-length protein will be needed to confirm that this region does not form a helical structure (Andreosso et al. 2018). The same study showed that a different partial peptide (CfTX-1_73-100_) in a predicted transmembrane motif displayed the expected structural and behavioral features for a membrane spanning region, which may be involved in the pore-forming mechanism (Andreosso et al. 2018). In the case of bioactivity, pore formation by JFTs was further supported by the observation of 12 nm (internal diameter) pores in human red blood cells exposed to JFTs (called porins) from *C. fleckeri* and *A. alata* (Yanagihara and Shohet 2012). Although cubozoan JFTs appear to share these putative α-helix and transmembrane regions, phylogenetic analysis of the protein sequences suggested a further split into two major groups: Type I (exemplified by CfTX-1/2) and Type II (exemplified by CfTX-A/B) (Brinkman et al. 2014). Antibodies against Type I and Type II toxins are not cross-reactive, suggesting some unique structural features distinguish each group (Brinkman et al. 2014).

Within functionally characterized cubozoan JFTs, there is clear variation in both intensity and specificity of their pore-forming properties. For instance, CfTX-A/B (Type II) toxins displayed at least 30 times more hemolytic activity compared with CfTX-1/2 (Type I); however, CfTX-1/2 induces rapid cardiovascular failure in rats compared with the relatively little cardiovascular activity disruption observed with similar concentrations of CfTX-A/B (Brinkman et al. 2014). The substantial reactivity of Type I toxins against vertebrate cardiac cells suggests that they play a key role in human envenomation, as illustrated by the intense cardiovascular issues observed in reported cases of *C. fleckeri* stings (Brinkman et al. 2014). The initial functional assays for CrTX-A, CaTX-A, and CqTX-A toxins indicated variable levels of hemolytic activity (EC_50_ ∼ 2, 70, 160 ng/ml, respectively) and lethality to crayfish (LD50 ∼ 5, 5–25, 80 μg/kg, respectively) (Nagai 2003), as well as cutaneous inflammation (likely leading to necrosis) and lethality in mice in the case of CrTX-A (LD_50_ ∼ 20 μg/kg) (Nagai, Takuwa-Kuroda, et al. 2000). Both Type II toxins display potent hemolytic activity consistent with findings from Type II toxins in *C. fleckeri*. The high potency in crayfish lethality assays may indicate a greater effect of Type II toxins on crustaceans and other major invertebrates, which are common prey items for cubozoans (Hamner et al. 1995; Lewis Ames and Macrander 2016). Although the Type I toxin CqTX-A appears to be comparatively less potent in both assays, *C. yamaguchii* stings have previously caused deaths from cardiac collapse (Fenner and Williamson 1996; Nagai et al. 2002; Nagai 2003), which is consistent with findings of Type I toxins in *C. fleckeri*.

Though JFTs were previously assumed to be specific to cubozoans, they have recently been identified in scyphozoans, such as *Aurelia* spp. (Rachamim et al. 2015; Gold et al. 2019), *Cassiopea xamachana* Bigelow, 1892 (Ames, Klompen et al. 2020), *Cyanea capillata* (Linnaeus, 1758) (Lassen et al. 2011), *Nemopilema nomurai* Kishinouye, 1922 (Li et al. 2014 as *Stomolophus meleagris*, Wang et al. 2018, but see Li et al. 2020), and *Chrysaora fuscescens* Brandt, 1835 (Ponce et al. 2016), and the hydrozoan *Hydra vulgaris* Pallas, 1766 (as *H. magnipapillata*) (Balasubramanian et al. 2012; Rachamim et al. 2015) (see additional examples in supplementary table S1, Supplementary Material online). Although a small number of predicted genes or transcripts of putative JFTs have been found in anthozoans (Rachamim et al. 2015; Gacesa et al. 2015; Surm et al. 2019; Klompen et al. 2020), no matching peptides have been identified using proteomic analyses of extracted crude venom or tentacle tissue from this clade, casting doubt that these are true toxin-coding genes. The apparent restriction of JFTs to medusozoan venoms resulted in the current nomenclature of “jellyfish toxins” (Jouiaei, Yanagihara, et al. 2015), though several different names have been used over the last two decades (table 1). The presence of JFT’s in other medusozoan species, in particular, those that are mild or harmless to humans, complicates the narrative that these toxins are solely responsible for the potent sting of cubozoans. More recently, it has been suggested that the potency of cubozoan venoms could be related to the following two factors: 1) the dominance (i.e., relatively high abundance) of JFT orthologs in the venom profile, as observed in the cubozoan *C. fleckeri* and 2) an expansion of JFT orthologs and, hypothetically, subsequent functional diversification (Brinkman et al. 2014, 2015).

**Table 1:** Past and Current Nomenclature for Jellyfish Toxin Family

References	Nomenclature of Protein Group
Klompen et al. (2020)	Jellyfish toxins
Surm et al. (2019)	Jellyfish toxins
Andreosso et al. (2018)	CfTX protein toxins
Podobnik and Anderluh (2017)	Three-domain Cry-like toxin
Jaimes-Becerra et al. (2017)	Jellyfish toxins
Lewis Ames et al. (2016), Ames and Klompen et al. (2020)	CaTX/CrTX toxin family
Ponce et al. (2016)	Box jellyfish toxins
Jouiaei, Sunagar, et al. (2015) and Jouiaei, Yanagihara, et al. (2015)	Jellyfish toxins
Brinkman et al. (2015)	CfTXs, CxTX
Rachamim et al. (2015)	CaTX-like
Brinkman et al. (2014)	CfTX-like
Yanagihara and Shohet (2012)	Porins
Badré (2014)	CaTX/CrTX toxin family
Anderluh et al. (2011)	Box jellyfish and jellyfish cytolytic toxins
Brinkman and Burnell (2007, 2008)	Box jellyfish toxins
Sher et al. (2005)	CaTX-like
Nagai (2003)	Box jellyfish toxin family

JFT gene duplication and subsequent alteration of function is consistent with previous studies that show venom genes can evolve through the duplication of physiologically important and conserved gene families, which undergo subfunctionalization (partition of ancestral function) or neofunctionalization (obtaining a new function) through various molecular evolutionary mechanisms (Sunagar et al. 2014). Previous work on anthozoans has shown that duplication plays an important role in the venom repertoire of this cnidarian group (Gacesa et al. 2015; Surm et al. 2019), and that duplicated toxins with similar or newly evolved functions may become differentially expressed in distinct tissues and/or across life stages (e.g., Surm et al. 2019). This general process of gene duplication followed by neo- or subfunctionalization has been shown exceptionally clearly in the starlet sea anemone *Nematostella vectensis*, Stephenson, 1935, including the NEP3 family across different nematocyte subpopulations (Columbus-Shenkar et al. 2018) and Nv1 family across various life stages and cell types (Sachkova et al. 2019). In particular, the Nv1 derived toxins Nv4 and Nv5 showed a dramatic change in function: while Nv1 is active against arthropod sodium channels, Nv4/Nv5 toxins are active against fish via an unknown receptor (Sachkova et al. 2019). In the case of cubozoan JFTs, whose ecological role is unknown but assumed to be for predation, the presence of multiple orthologs could reflect a shift in cubozoan behavior toward hunting and a shift in diet toward vertebrate prey (e.g., fish) (Lewis Ames and Macrander 2016). Thus, neo- and/or subfunctionalization of duplicated toxin genes might also inform the evolution of medusozoan ecological interactions, such as dietary changes or defense against specific predators.

The emerging evidence that the JFT family evolved through gene duplication and subsequent functional diversification makes this family an ideal model to investigate the molecular evolutionary mechanisms underlying the evolution of ecological interactions across Medusozoa, including groups with extreme toxicity. Previous work has shown that genes coding for toxins of early diverging lineages of venomous organisms experience greater purifying selection than those of more recently diverged venomous clades (Sunagar and Moran 2015), which has also been suggested in more focused studies on cnidarian venoms (Rachamim et al. 2015, Jouiaei, Sunagar, et al. 2015). Jouiaei, Sunagar, et al. (2015) specifically tested the influence of selection on a subset of JFTs and found that this family was influenced by negative (purifying) selection, though one test suggested up to 17 sites across these sequences were influenced by episodic periods of positive (diversifying) selection. On the one hand, the high level of structural conservation across diverse PFT families to create pores suggests that the observed purifying selection in JFTs is likely related to the preservation of pore-forming activity (Jouiaei, Sunagar, et al. 2015). However, the diversity of JFT specificity toward different cell targets implies diversifying selection pressure on functional regions or sites responsible for cell specificity (Brinkman et al. 2014; Podobnik and Anderluh 2017). A better understanding of JFT gene family evolution across medusozoans could not only provide a framework for understanding the variable potency of these toxins, but could also more broadly explain why some cubozoans are such dangerous stingers for humans. The structural novelty of these PFTs (as well as other cnidarian toxins) could lead to the development of novel therapeutic drugs or other beneficial research tools (Mariottini and Pane 2013; Herzig et al. 2020).

Here, we explored all available cnidarian transcriptome data to identify and annotate putative JFT genes and classified them within a phylogenetic framework. Using our well-supported gene tree, we investigated the molecular evolution of these genes to determine the potential role of positive selection for various subgroups. Additionally, we investigated temporal and spatial variation in the expression of JFTs between functionally distinct tissues across several species.

## Results

### Identification and Annotation of Novel Candidate JFTs

**Fig. 2.**
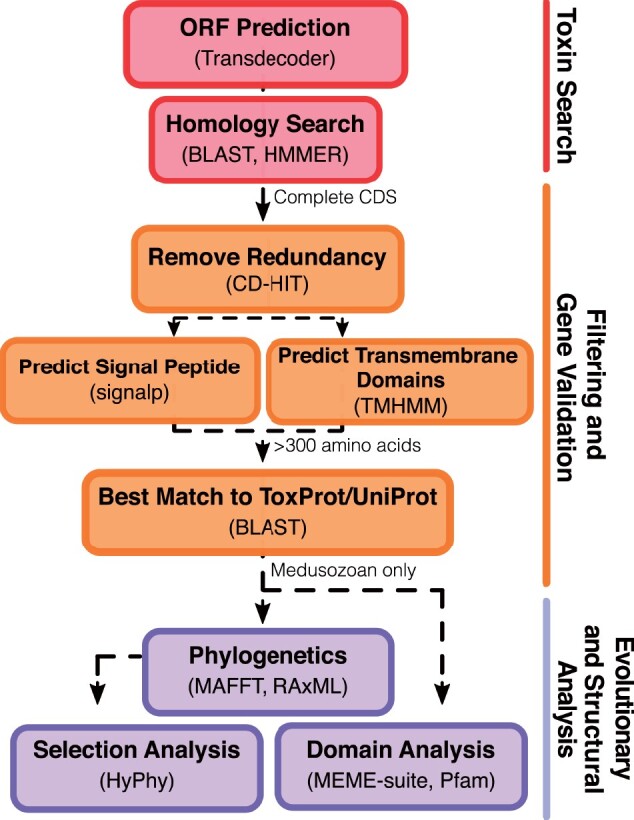
Custom bioinformatic pipeline used in this study to identify and filter novel JFT-like genes within publicly available transcriptome data.

From an initial search of predicted open readings frames (ORFs) from 147 transcriptome assemblies (supplementary table S2, Supplementary Material online), we captured 293 total potential JFT candidate genes after removing redundant sequences (supplementary table S3, Supplementary Material online), and before our stringent filtering step (see the custom bioinformatic pipeline described in fig. 2). The final data set consisted of 124 sequences, of which 111 are novel putative JFTs from over 20 species of medusozoans selected for phylogenetic analyses (supplementary table S3, Supplementary Material online). JFT-like toxin sequences that have previously been reported for *C. capillata* (Liu et al. 2015, but see Wang et al. 2018) and *Nemopilema nomurai* (Li et al. 2014, Wang et al. 2018) were not included in our analyses as they were recovered as partial sequences and did not meet the more stringent criteria of our annotation pipeline. Similarly, we only retained two complete toxins out of the 15 previously reported by Brinkman et al. (2015) in *C. fleckeri* (not including the references JFT sequences derived from *C. fleckeri*). Several conserved motifs were identified among the JFT set (111 novel toxins + 13 references JFTs) that are similar to conserved regions described in previously reported JFTs from cubozoans (Brinkman et al. 2014), including a previously identified predicted transmembrane domain region (supplementary fig. S1 and table S4, Supplementary Material online). However, no other distinguishable domain of known function shared between all JFT sequences was detected using MEME-suite, and outside the putative α-helical region and transmembrane domain, no distinct domain or domain architecture has been reported for JFTs, further validated by our own search of these 124 sequences against the Pfam database (supplementary table S4, Supplementary Material online).

We identified 15 putative JFTs from ten species of anthozoans that passed our filtering criteria, including four recently reported sequences from three ceriantharian (tube anemones) species (Klompen et al. 2020). Preliminary phylogenetic analyses did not recover stable placement for the anthozoan sequences, with low bootstrap support for many of the clades and little discernable taxonomic patterns. When Cry toxins were included as the root, all anthozoan sequences except the stony corals *Madracis auretenra* Weil and Coates, 2007 and *Stylophora pistillata* Esper, 1797 were weakly clustered (bootstrap support = 42) as a sister-clade to JFT-1 (see below) (bootstrap support = 36); when Cry toxins were not included and the sequences from *M. auretenura* and *S. pistillata* were used as the root the anthozoan cluster was better resolved (bootstrap = 100), but the clade remained weakly placed as sister to JFT-1 (bootstrap support = 27) and the cerianthid sequence from *Pachycerianthus* cf. *maua* (Carlgren, 1900) was very weakly placed in the same clade as the *Atolla vanhoeffeni* Russell, 1957 sequences (bootstrap support = 11) (supplementary figs. S2 and S3, Supplementary Material online). To the best of our knowledge, predicted JFT-like genes from anthozoans have only been identified via genomic (e.g., Gacesa et al. 2015) and transcriptomic studies (e.g., Rachamim et al. 2015; Surm et al. 2019; Klompen et al. 2020), with no JFT-like peptides isolated from proteomic analyses that included anthozoan venoms (e.g., Rachamim et al 2015; Surm et al. 2019). Given that current evidence does not support anthozoans possessing JFT-like toxins in their venoms, our study focused on medusozoan JFT-like candidates.

### Phylogenetic Analysis Reveals Two Main Groups of JFTs

**Fig. 3.**
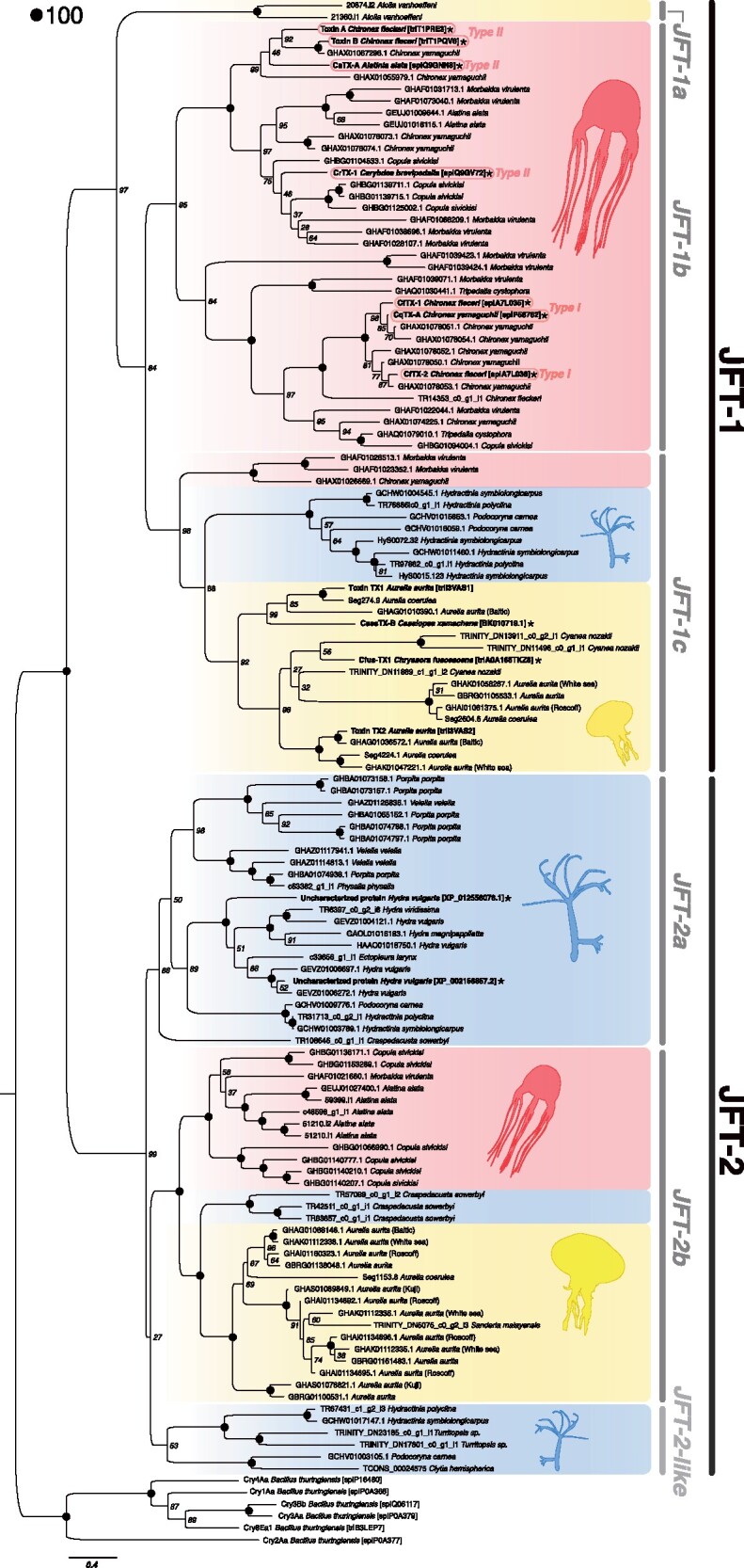
Maximum likelihood phylogeny of medusozoan JFT genes in this study. Phylogeny was constructed using RAxML and 500 rapid bootstrap replicates (all shown). Bolded sequences are previously identified JFTs or JFT-like sequences. Stared sequences (*) indicate those added based on proteomic evidence. Blue = Hydrozoa, yellow = Scyphozoa, red = Cubozoa. Maximum bootstraps support values (100) are indicated as black dots.

The maximum likelihood (ML) gene phylogeny partitioned JFTs into two major groups, referred hereafter as JFT-1 (bootstrap support = 97) and JFT-2 (bootstrap support = 99), respectively (fig. 3), which is consistent with previous studies that had less extensive gene sampling and less stringent filtering criteria (Brinkman et al. 2015; Lewis Ames et al. 2016).

Within JFT-1, we recovered three separate clades named JFT-1a, -1b, and -1c with high support values (100, 95, and 98, respectively; fig. 3). JFT-1a represents two paralogs from *A. vanhoeffeni*, the only representative coronate scyphozoan. JFT-1b contains a large set of cubozoan-only JFT genes, including two subclades that roughly correspond to the Type I and Type II subfamilies previously described by Brinkman et al. (2014) (fig. 3). All previously described cubozoan toxins that have biochemically supported evidence of a pore-forming activity (e.g., CaTX-A, CrTX-1, CqTX-1, CfTX-A, CfTX-B, CfTX-1, and CfTX-2) were recovered within the JFT-1b clade. JFT-1c contains sequences from cubozoans, hydrozoans, and scyphozoans. The three cubozoan JFT-1c sequences are from *Morbakka virulenta* (Kishinouyea, 1920) (*n* = 2) and *C. yamaguchii* (*n* = 1), and both species also have multiple orthologs in other JFT clades across the tree (fig. 3). The scyphozoans in JFT-1c included a previously described *Chrysaora fuscescens* toxin, Cfus-TX1 (Ponce et al. 2016), two toxins from the polyps of *Aurelia aurita* (Linnaeus, 1785) (Toxin 1 and Toxin 2) (The UniProt Consortium 2019), and a recently described toxin from *Cassiopea xamachana* (CassTX-B) (Ames and Klompen et al. 2020), as well as additional sequences from *Aurelia* spp. and *C. nozakii*.

JFT-2 was divided into three groups: 1) a strongly supported clade (bootstrap support = 86) of exclusively hydrozoan sequences (JFT-2a); 2) a strongly supported clade (bootstrap support = 100) containing well-supported subclades of hydrozoans, scyphozoans and cubozoans sequences (JFT-2b); and 3) a weakly supported (bootstrap support = 53) clade of hydrozoan sequences (JFT-2-like) sister to JFT-2b (fig. 3). JFT-2b is divided into two strongly supported subclades (bootstrap support = 100): a cubozoan-only clade that includes putative toxins from *A. alata*, *Copula sivickisi* (Stiasny, 1926), and a single *M. virulenta* sequence and a scyphozoan-only clade represented by several *Aurelia* spp. and a single *Sanderia malayensis* Goette, 1886 sequence rooted by sequences from the trachyline hydrozoan *Craspedacusta sowerbyi* Lankester, 1880. In contrast to JFT-1 where hydrozoan representatives were restricted to hydractiniids, 12 species belonging to 6 hydrozoan families were represented in JFT-2. Proteomic-level isolation of JFT-2 toxins are limited to two peptides from *H. vulgaris* identified within the nematocyst proteome (Balasubramanian et al. 2012), both of which fall in JFT-2a.

### Influence of Gene-Specific Episodic Positive Selection on Cubozoan Toxins

**Fig. 4.**
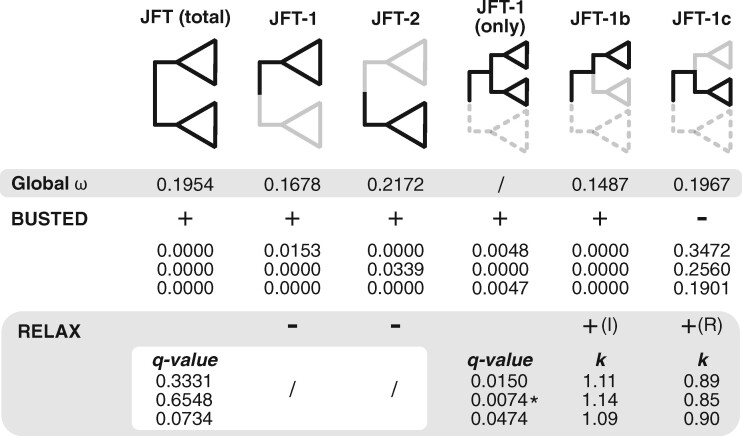
Summary of global *dN*/*dS*, BUSTED, and RELAX results using HyPhy. Global *dN*/*dS* values shown for each focal clade. FDR-corrected *P*-values (*q*-values) are shown for the three subsets in both BUSTED and RELAX tests. For RELAX tests, q-values shown are the same given the FDR-correction was only for two tests (JFT-1 vs. JFT-1, JFT-1b vs. JFT-1c); for example, *q*-values in the white box are the same for both JFT-1 versus JFT-2 and JFT-2 versus JFT-1. For significant RELAX tests (*q*-value < 0.05) (in gray), *k* parameters are shown. For *k* > 1, selection strength has been intensified, and for *k* < 1, selection strength has been relaxed. *I* = intensification of selection. *R* = relaxation of selection. (*) indicates RELAX tests run on Datamonkey server v2 (accessed January 2021; Weaver et al. 2018). Slashes indicate no data shown. Uncorrected *P*-values and *k* parameters for all RELAX tests are shown in supplementary table S5, Supplementary Material online.

Overall, the *dN*/*dS* ratio for sequences within the phylogeny was consistently <1, suggesting that the JFT toxin family is broadly under negative selection (fig. 4). The *dN*/dS ratio is lower for JFT-1 (ω = 0.1678) compared with JFT-2 (ω = 0.2172), and JFT-1b (ω = 0.1487) compared with JFT-1c (ω  =  0.1967), suggesting that negative selection may be acting more strongly on these cubozoan-dominant clades. However, to explicitly test for positive selection, we used gene-specific and branch-specific (i.e., branch-site) tests as implemented via HyPhy (Kosakovsky Pond et al. 2020), with the hypothesis that cubozoan-specific JFTs have undergone episodic positive selection across specific sites compared with other taxonomic groups.

Using the Branch-Site Unrestricted Statistical Test for Episodic Diversification (BUSTED) test, we found evidence of episodic gene-wide positive selection across the cubozoan-only JFT-1b clade when compared with JFT-1c (FDR-corrected *P*-value = 0.000 across all three subsets) but not when JFT-1c is compared with JFT-1b (FDR-corrected *P*-value ≥0.1901). This indicates that at least one site on at least one branch of the cubozoan-dominated JFT-1b subclade has undergone positive selection compared with the scyphozoan and hydrozoan JFT-1c branches and genes. Using RELAX, we found selection was intensified in JFT-1b versus JFT-1c in all three subsets (FDR-corrected *P*-value ≤0.0474; fig. 4), suggesting that the cubozoan JFT-1b lineages have undergone episodic positive selection followed by intensification of selection. Using BUSTED and RELAX on the three larger clades (i.e., JFT-1 vs. JFT-2, JFT-2 vs. JFT-1, and JFTs overall) indicated all of these groups display a degree of gene-wide positive selection (FDR-corrected *P*-values ≤0.0339; fig. 4) but not relaxation (or intensification) of selection (FDR-corrected *P*-values ≥0.0734; fig. 4), which further suggests that distinct selection forces are acting on the cubozoan JFT-1b branches.

Adaptive branch-site random effects likelihood (aBSREL; Smith et al. 2015) found evidence for episodic positive selection across several branches throughout the phylogeny (supplementary figs. S4–S6, Supplementary Material online). However, these tests were inconsistent between subsets and typically resulted in terminal sequences. Both site-based tests also found sites under the influence of pervasive positive selection (four sites in FUBAR, posterior probability >0.9) and episodic positive selection (18 sites in MEME, FDR-corrected *P*-value <0.05) (supplementary table S5, Supplementary Material online).

**Table 2:** Reference Jellyfish Toxins Used for Searches and Annotation

Name	Species	Class	Len.	UniProt Acc.	NCBI Acc.
CrTX-A	*Carybdea brevipedalia* (as *C. rastonii*)	Cubozoa	450	Q9GV72	AB015878.1
CaTX-A	*Alatinia alata* (as *C. alata*)	Cubozoa	463	Q9GNN8	BAB12727.1
CqTX-A	*Chironex yamaguchii* (as *Chiropsalmus quadrigatus*)	Cubozoa	462	P58762	AB045319.1
CfTX-1	*C. fleckeri*	Cubozoa	456	A7L035	ABS30940.1
CfTX-2	*C. fleckeri*	Cubozoa	462	A7L036	ABS30941.1
CfTX-A	*C. fleckeri*	Cubozoa	454	T1PRE3	AFQ00677.1
CfTX-B	*C. fleckeri*	Cubozoa	461	T1PQV6	AFQ00676.1
TX-like	*C. fleckeri*	Cubozoa	296	W0K4S7	AHG06297.1
Cytotoxin A—Isoform 1	*Malo kingi*	Cubozoa	457	D2DRC0	ACX30670.1
Cytotoxin A—Isoform 2	*M. kingi*	Cubozoa	453	D2DRC1	ACX30671.1
Toxin TX1	*Aurelia aurita*	Scyphozoa	486	3VAS1	AFK76348.1
Toxin TX2	*A. aurita*	Scyphozoa	452	3VAS2	AFK76349.1
CfusTX-1	*Chrysaora fuscescens*	Scyphozoa	457	A0A165TKZ8	AMY95568.1

### JFTs Display Differential Expression between Life Stages, Tissues, and Cell Types

We explored publicly available RNA-seq data sets (with replicates) for several medusozoan species, including for the hydrozoans *Hydractinia symbiolongicarpus*, Buss and Yund 1989 (Sanders et al. 2014), *Podocoryna carnea*, Sars 1946 (Sanders and Cartwright (2015a), and *Clytia hemisphaerica* (Linnaeus 1767) (Leclère et al. 2019), as well as the scyphozoan *Aurelia coerulea*, von Lendenfeld 1884 (Gold et al. 2019 as *Aurelia* sp. 1) to survey if JFT sequences uncovered by our study were considered upregulated or downregulated in similar tissue types or life stages (table 4). In medusae-bearing species (*Podocoryna*, *Clytia*, and *Aurelia*), JFTs were upregulated in developing or adult medusae. The colonial hydrozoan *H. symbiolongicarpus* does not have a medusa stage but does display a division of labor of functionally and morphologically distinct polyp types: gastrozooids (prey capture, digestion), gonozooids (reproduction, no mouth), and dactylozooids (prey capture, no mouth). Four different JFT paralogs display unique expression profiles in the different polyps; two are upregulated in gastrozooids (GCHW01004545.1, GCHW01017147.1), one is upregulated in dactylozooids (GCHW01011460.1), and the fourth is downregulated in dactylozooids (GCHW01003789.1), with all comparisons made with respect to the other polyp types (Sanders et al. 2014; Sanders and Cartwright (2015b). Two other JFTs were identified in the *H. symbiolongicarpus* genome that are also found in JFT-1 but were not detected in this particular transcriptomic study. For *Hydra*, all JFTs within the single-cell data set were upregulated in cell lineages associated with nematoblasts (immature nematocysts), including some specifically associated with stenotele development (Siebert et al. 2019).

## Discussion

Our survey of JFTs in cnidarians using publicly available data reveals that they encompass considerable diversity across medusozoans. Phylogenetic analysis of JFTs recovers distinct taxonomic patterns across clades (fig. 3 and table 3), which enables us to make predictions about functional variation observed within this toxin family. In total, we assigned 111 complete sequences to this family, a significant increase from nonpartial toxins reported in previous studies (∼30; supplemental table S1, Supplementary Material online), even despite our more stringent identification criteria. We identified JFT-like sequences in many species that are considered harmful stingers to humans, including (but not limited to) the siphonophore *Physalia physalis*, Linnaeus 1758 (Tamkun and Hessinger 1981), and the scyphozoans *C. nozakii* (Feng et al. 2010) and *S. malayensis*, as well as several species that are considered harmless or weak stingers to humans, including several hydrozoan species (table 3). Our ML phylogeny recovered two major clades, one dominated by cubozoans (JFT-1) and the other by hydrozoans and scyphozoans (JFT-2; fig. 3). This topology is consistent with the two clades observed in previous studies (e.g., Brinkman et al. 2015), but our sampling vastly increases species representation, particularly within the JFT-2 group.

**Table 3:** Number of Annotated JFT Orthologs for Each Taxa Sorted by Subclade

Species	Order	Total	JFT-1a	JFT1-b	JFT-1c	JFT-2a	JFT-2b	JFT-2-like
Cubozoa								
* Chironex fleckeri*	Chirodroppida	5	0	5	0	0	0	0
* C. yamaguchii*	Chirodroppida	12	0	11	1	0	0	0
* Alatinia alata*	Carybdeida	8	0	3	0	0	5	0
* Carybdea brevipedalia*	Carybdeida	1	0	1	0	0	0	0
* Copula sivickisi*	Carybdeida	11	0	5	0	0	6	0
* Morbakka virulenta*	Carybdeida	12	0	9	2	0	1	0
* Tripedalia cystophora*	Carybdeida	2	0	2	0	0	0	0
Scyphozoa								
* Atolla vanhoeffeni*	Coronata	2	2	0	0	0	0	0
* Aurelia aurita*	Ulmaridae (Disco)	6	0	0	3	0	3	0
* A. aurita* (*Baltic*)	Ulmaridae (Disco)	3	0	0	2	0	1	0
* A. aurita* (*Kuji*)	Ulmaridae (Disco)	2	0	0	0	0	2	0
* A. aurita* (*Roscoff*)	Ulmaridae (Disco)	5	0	0	1	0	4	0
* A. aurita* (*White sea*)	Ulmaridae (Disco)	5	0	0	2	0	3	0
* A. coerulea* (*Aurelia* sp.1)	Ulmaridae (Disco)	4	0	0	3	0	1	0
* Cassiopea xamachana*	Rhizostomeae	1	0	0	1	0	0	0
* Cyanea nozakii*	Cyanidae	3	0	0	3	0	0	0
* Chrysaora fuscescens*	Pelagiidae	1	0	0	1	0	0	0
* Sanderia malayensis*	Pelagiidae	1	0	0	0	0	1	0
Hydrozoa								
* Craspedacusta sowerbyi*	Trachylina	4	0	0	0	1	3	0
* Velella velella*	Capitata	3	0	0	0	3	0	0
* Porpita porpita*	Capitata	6	0	0	0	6	0	0
* Hydractinia polyclina*	Filifera (III)	4	0	0	2	1	0	1
* H. symbiolongicarpus*	Filifera (III)	6	0	0	4	1	0	1
* Podocoryna carnea*	Filifera (III)	4	0	0	2	1	0	1
* Turritopsis* sp.	Gonoproxima (IV)	2	0	0	0	0	0	2
* Clytia hemispherica*	Leptothecata	1	0	0	0	0	0	1
* Physalia physalis*	Siphophora	1	0	0	0	1	0	0
* Hydra viridissima*	Aplanulata	1	0	0	0	1	0	0
* H. vulgaris (H. magnipappilatta*)	Aplanulata	7	0	0	0	7	0	0
* Ectopleura larynx*	Aplanulata	1	0	0	0	1	0	0

Within JFT-1, we recovered a high number of sequences within the cubozoan-specific clade; roughly twice as many genes per species are represented in the JFT-1b group than the JFT-1c clade. This suggests there has been an extensive expansion of JFT-1 genes within Cubozoa through gene duplication, specifically in the JFT-1b subclade (fig. 3), consistent with previous assumptions that an expansion of the JFT gene repertoire through gene duplication is associated with the comparatively higher potency of cubozoans venoms (Brinkman et al. 2014, 2015). This assertion assumes that cubozoan toxins have been influenced by positive (diversifying) selection driving increased potency, potentially due to a shift in the use of their venoms toward capturing vertebrate prey (see below). Interestingly, the carybdeid species *M. virulenta* (family Carukiidae) and *A. alata* (family Alatinidae), as well as the chirodropid *C. yamaguchii* (family Chirodropida) display some of the most extensive gene duplication patterns in the pore-forming JFT-1b clade, which may explain why these animals are notorious for their toxicity to humans (Bentlage et al. 2010). Our analyses detected intensification of selection and gene-wide positive selection within JFT-1b, suggesting functional changes of duplicated JFTs in these dangerous cubozoan species could be a driver for the increased toxicity.

Based on the taxonomic distribution in our phylogeny and our current functional understanding of specific JFTs, an interesting pattern of hypothesized cytolytic function across the JFT family, in particular within the JFT-1 clade, emerges. All functionally characterized cubozoan toxins are found within JFT-1b (Nagai, Takuwa-Kuroda, et al. 2000; Nagai, Takuwa-Kuroda, Nakao, et al. 2000; Chung et al. 2001; Nagai et al. 2002; Brinkman and Burnell 2008; Yanagihara and Shohet 2012; Brinkman et al. 2014) and are subdivided into two subclades that appear to correspond to previously characterized Type I and Type II toxins (fig. 3; Brinkman et al. 2014). The clade corresponding to Type I, which is thought to have greater cardiotoxic activity compared wirh Type II, includes 7/11 paralogs from *C. yamaguchii*, a dangerous stinger that is responsible for many fatalities in Japan and the Philippines (Fenner and Williamson 1996). However, this clade also includes toxins from *Tripedalia cystophora* Conant, 1897, which is not known to have a dangerous sting to humans, potentially due to the small size of its nematocysts (Orellana and Collins 2011). Several orthologs from *C. sivickisi*, *M. virulenta*, and *A. alata*, as well as the two *Chironex* species are found in the Type II group, which is considered to have greater hemolytic bioactivity (Brinkman et al. 2014) though hemolytic activity has not been reported as a feature of cubozoan envenomation in humans (Brinkman et al. 2014). Thus, in addition to early duplication, the functional partitioning (i.e., potency variation) of JFT-1b genes suggests that these toxins may have undergone either neofunctionalization or subfunctionalization. Further study would be needed on the spatial and temporal expression of JFT-1b toxins in species where both Type I and Type II JFTs genes are identified (e.g., *C. yamaguchii*, *M. virulenta*, *C. sivickisi*) to determine which of these mechanisms is the most likely.

Interestingly, all of the scyphozoan species that possess JFT-1 genes have been known to cause painful envenomation to humans and/or are associated with cytolytic and hemolytic effects (Radwan et al. 2001; Feng et al. 2010; Kawabata et al. 2013; Ponce et al. 2015). The presence of several paralogs of toxin genes in some of these species could increase the toxicity of their venom and may result in more harmful stings to humans, such as in the lion’s mane jellyfish *C. nozakii* (Feng et al. 2010; Li et al. 2014). However, we also found multiple JFT paralogs in species considered much less potent stingers, such as the upside-down jellyfish *Cassiopea*, moon jellyfish *Aurelia*, and several species of hydrozoans. Even the less potent venoms of *Cassiopea* and *Aurelia* have some cytolytic activity that can cause irritation in the event of envenomation (Radwan et al. 2001). Curiously, the only hydrozoan representatives within the JFT-1 clade are from the family Hydractiniidae (*H. symbiolongicarpus*, *H. polyclina*, *Podocoryna carnea*), whereas many other hydrozoan species were represented in JFT-2, in particular JFT-2a. It is unclear why only hydractiniids have representatives within the JFT-1 clade, and what such representation means in terms of venom function and potency. Put together, this suggests that all JFT-1 sequences display broad cytolytic function with some specificity toward hemolytic activity.

None of the JFT-2 representatives have been characterized at a functional level, and it is unclear whether these toxins are 1) less potent than JFT-1 toxins, 2) have distinct cell targets, and/or 3) display any cytolytic bioactivity. Several species that only possess representatives from the JFT-2 clade have previously characterized hemolytic toxins, such as *P. physalis* (Tamkun and Hessinger 1981), or cytotoxic bioactivity in their venoms, such as *Velella velella* (Linnaeus 1758) (KilLi et al. 2020). Our MEME-suite analyses detected a similar domain structure within the majority of the JFTs in our study (JFT-1 and JFT-2) that corresponds to a previously proposed transmembrane region (supplementary fig. S1, Supplementary Material online), which has been experimentally determined to be functional in a representative CfTX-1 peptide and may be an important structural feature in pore formation (Andreosso et al. 2018). Based on their phylogenetic distribution, and the presence of this putative transmembrane region, it is likely that all JFT-1 toxins, and possibly all JFTs, display cytotoxic effects through a pore-forming mechanism. Further studies regarding the function of JFTs outside Cubozoa (especially when multiple paralogs are present) will provide greater insight into the ecological role played by these diverse medusozoans.

Our selection analyses suggest that the JFT family overall is under the influence of purifying (negative) selection across the JFT phylogeny and within individual JFT clades (fig. 4) according to *dN*/*dS* calculations, which is consistent with previous studies that had lower taxonomic sampling for JFTs (Jouiaei, Sunagar, et al. 2015; Surm et al. 2019). Negative selection pressure can easily be explained by the functional constraint of these proteins to create cell membrane pores (Anderluh and Lakey 2008; Peraro and van der Goot 2016), and negative selection is generally observed across toxin families of early diverging venomous animals (Sunagar et al. 2014; Sunagar and Moran 2015). However, our expectation was that while purifying, selection may be occurring broadly across JFT sequences, a subset of genes (i.e., sites) along a subset of lineages may be undergoing positive selection, referred to as episodic positive selection (Murrell et al. 2012). BUSTED analysis detects if episodic positive selection has occurred in at least one site on at least one branch within a set of target branches, but this method cannot establish specific sites under selection nor does it imply the same site has been influenced by episodic positive selection across all test branches (Murrell et al. 2015). BUSTED detected gene-specific positives selection in all larger clades (JFT-1, JFT-2, JFT-total) as well as JFT-1b compared with JFT-1c, but when JFT-1c was compared with JFT-1b, no positive selection was detected. The RELAX test does not explicitly test for positive selection, but instead estimates the strength of natural selection of a set of test branches compared with another (Wertheim et al. 2015). Although no evidence of relaxed selection was found in the more broadly tested JFT-1 and JFT-2 groups, we identified intensification of selection in all three subsets of sequences where the cubozoan-dominant JFT-1b was compared with JFT-1c (mainly hydrozoan and scyphozoan representatives). Although it is unclear whether the detected regions in our analysis are functionally relevant, our results suggest that specific yet undetermined regions within cubozoan JFT-1b toxins experienced intensification of selection as well as episodic positive selection that has not occurred (or is otherwise not detectable) in JFT-1c toxins. We interpret this discrete (i.e., localized) positive selection of potentially functional regions as likely concomitant with increased potency and/or specificity of the JFT-1b family. Overall, these patterns suggest that JFTs have undergone a complex history of evolutionary selective pressures, including wide-spread purifying selection, gene-specific episodic positive selection on a particularly toxic clade of cubozoan sequences, as well as a few instances of episodic positive selection across certain taxa.

Notably, both site-specific tests detected sites undergoing positive selection: 4 sites under pervasive selection according to FUBAR and 18 sites under episodic selection based on calculated FDR-corrected *P*-values from MEME (supplementary table S5, Supplementary Material online), but neither of these tests indicate the specific lineages where these sites are undergoing positive selection. To our knowledge, no available information exists on the functionally important residues in JFT sequences, which means these site-specific results should be regarded as exploratory. Additional structural research complimented by functional tests to manipulate specific residues in these JFTs are the best way to resolve the question of which residues may be the most critical to bioactivity, and it is our hope that this work will provide a foundation for such studies.

The episodic positive selection signatures together with high levels of gene expansion detected within the cubozoan sequences in JFT-1b may be related to the predation patterns of these species. Venom is an important evolutionary mechanism in an animal’s ability to subdue and consume specific prey items, and as such several studies have explored the coevolution of venom composition and diet (e.g., Dutertre et al. 2014). Several cubozoan species are well known for being active fish hunters, thus the potency of JFTs in cubozoans may reflect selection pressure for an increased ability to capture and subdue fast, vertebrate prey (Courtney et al. 2015; Lewis Ames and Macrander 2016), rendering these toxins subsequently dangerous and potentially fatal to humans. Interestingly, we identified two JFT-1b homologs in *T. cystophora*, a cubozoan that is not as dangerous a stinger for humans as other cubozoan species and appears to primarily feed upon crustacean copepods rather than fish or other vertebrate prey (Stewart 1996). Conversely, several species that lack representatives from the JFT-1b clade are also known to be toxic to vertebrates, including the scyphozoans *C. nozakii* (Feng et al. 2010) and *C. fuscescens* (Ponce et al. 2016). Instead, these relatively potent stingers have representatives in the JFT-1c clade along with less toxic species such as the moon jelly *Aurelia* and the upside-down jellyfish *Cassiopea*, although mucus released from *Cassiopea* is able to sting fish (Stoner et al. 2014) as well as humans (Ames and Klompen et al. 2020). This suggests that not all JFT-1 proteins are potent toxins or, alternatively, are not abundant enough within the delivered venom cocktail of these specific species to cause severe medical damage to humans. The function of JFT-2 representatives is even more enigmatic given no toxin within this group has been functionally characterized to date. JFT-2 is populated by several hydrozoan species ranging from weak to potent stingers, and other than *P. physalis*, none are known to prey on fish. It should be noted that our understanding of cubozoan diets, and cnidarians in general, is relatively limited and itself requires additional study (Lewis Ames and Macrander 2016). The relationship between JFT potency and specificity to vertebrate predation (or defense) will require additional sampling, functional studies, and greater focus on understanding the ecological interactions of these species, in particular within noncubozoan medusozoans.

Unlike other venomous animals, cnidarians have a decentralized venom system spread across their tissues. Thus, we can predict distinct expression profiles of various venom components (i.e., toxin orthologs) in different tissues to interpret specific ecological functions (e.g., Columbus-Shenkar et al. 2018; Sachkova et al. 2019; Surm et al. 2019). In the case of JFTs, the high number of gene duplication events within several species suggests the possibility for biased gene expression across functionally different tissues.

Overall, we found that JFT paralogs have distinct expression profiles that are spatially and temporally distributed across several biological levels (table 4): 1) distinct life cycle stages of several species, 2) distinct functional tissues within the colonial hydroid *Hydractinia*, and 3) distinct stinging cell types within *Hydra*. In comparisons of life stages where developing medusae, juvenile medusae, and/or mature medusae stages were available, all relevant species (i.e., *Podocoryna*, *Clytia*, and *Aurelia*) displayed upregulated JFT-expression in the these specific tissues compared with the earlier developmental stages such as planulae or polyps. This suggests that JFTs are predominantly used within the pelagic environment (e.g., Underwood and Seymour 2007), which could be a reflection of the distinct lifestyle of the medusa stage, including a wide range of diets and hunting strategies across species as well as the variety of predators in the pelagic realm. For instance, cassiosomes, stinging-cell structures released in the mucus of medusae of *Cassiopea*, contain peptides corresponding to JFT toxins. Cassiosome-laden mucus is assumed to assist with predation of zooplankton but has also been reported to deter fish (Stoner et al. 2014) and is associated with a stinging sensation in humans (Ames and Klompen et al. 2020). Within the functionally distinct polyp types of *Hydractinia*, the upregulated dactylozooid toxin and one of the gastrozooid toxins are in clade JFT-1, which suggests that both may display cytolytic function. Given both of these polyp types are specific to prey capture, it may indicate that JFTs are important to this key ecological role. The function of the two other toxins in JFT-2, one upregulated in the gastrozooid and one downregulated in the dactylozooid, is less clear, but they may be involved in digestion or defense rather than prey capture. Previous studies on the cubozoan *A. alata* showed some JFTs are expressed outside the nematocytes (Nagai, Takuwa-Kuroda, Nakao, et al. 2000) and within the gastric cirri (Lewis Ames et al. 2016), implying a role in digestion, though both studies proposed that JFT toxin synthesis may be occurring outside the nematocysts before being delivered to the mature stinging cell organelle (also see Lewis Ames and Macrander 2016). In the single-cell data set for *Hydra* (Siebert et al. 2019), all JFTs localized to nematoblasts, confirming that these are true toxins utilized by this species. Furthermore, some JFTs were upregulated in cell lineages specific to stenoteles, a type of penetrant nematocyst used for prey capture (Kass-Simon and Scapppaticci 2002). Overall, the distinct tissue profiles between JFT paralogs within each of these species suggests that JFTs have evolved in interesting and previously underappreciated ways across Medusozoa. Furthermore, these toxins may possess many diverse functional roles within the venom repertoires across other medusozoan species (Schendel et al. 2019), given that the group displays significant developmental (polyp vs. medusa, coloniality vs. solitary, etc.) and ecological diversity (variation is size and habitat, prey type and predation, symbiosis, etc.) (Kayal et al. 2018; Ashwood et al. 2020; Surm and Moran 2021).

**Table 4:** Summary of Expression Data of JFTs Compiled from Published Studies

Species	Tissue	Relative Diff. Expression	Transcript	Clade	References
*Hydractinia symbiolongicarpus*	Gastrozooid	Up	GCHW01004545.1	JFT-1c	Sanders et al. (2014, 2015b)
*H. symbiolongicarpus*	Dactylozooid	Up	GCHW01011460.1	JFT-1c	Sanders et al. (2014), Sanders and Cartwright (2015b)
*H. symbiolongicarpus*	Gastrozooid	Up	GCHW01017147.1	JFT-2-like	Sanders et al. (2014), Sanders and Cartwright (2015b)
*H. symbiolongicarpus*	Dactylozooid	Down	GCHW01003789.1	JFT-2a	Sanders et al. (2014), Sanders and Cartwright (2015b)
*Podocoryna carnea*	Budding polyp	Up	GCHV01016059.1	JFT-1c	Sanders and Cartwright (2015a,b)
*P. carnea*	Medusa	Up	GCHV01015653.1	JFT-1c	Sanders and Cartwright (2015a,b)
*P. carnea*	Budding polyp	Up	GCHV01009776.1	JFT-2a	Sanders and Cartwright (2015a, b)
*Clytia hemispherica*	Gonozooid; Medusa	Up	TCONS_00024575	JFT-2-like	Leclère et al. (2019)
*Aurelia coerulea*	Ephyra; Juvenile Medusa	Up	Seg274.9	JFT-1c	Gold et al. (2019)
*A. coerulea*	Early Strobila	Up	Seg2604.6	JFT-1c	Gold et al. (2019)
*A. coerulea*	Ephyra; Juvenile Medusa	Up	Seg4224.1	JFT-1c	Gold et al. (2019)
*Hydra vulgaris*	Nematoblast, stenotele	Up	HAAC01018750.1	JFT-2a	Siebert et al. (2019)
*H. vulgaris*	Nematoblast	Up	GAOL01016183.1	JFT-2a	Siebert et al. (2019)
*H. vulgaris*	Nematoblast, stenotele	Up	GEVZ01006697.1	JFT-2a	Siebert et al. (2019)
*H. vulgaris*	Nematoblast, stenotele	Up	GEVZ01004121.1	JFT-2a	Siebert et al. (2019)
*H. vulgaris*	Nematoblast	Up	GEVZ01006272.1	JFT-2a	Siebert et al. (2019)
H. vulgaris*	Nematoblast, stenotele	Up	XP_002156857.2*	JFT-2a	Siebert et al. (2019)
H. vulgaris*	Nematoblast	Up	XP_012556076.1*	JFT-2a	Siebert et al. (2019)

Note.—Transcripts labeled with (*) associated with peptides identified in Balasubramanian et al. (2012).

One of the caveats of our study is that our search methods depended on queries with previously described JFTs, most of which are from cubozoans. This suggests that our search method likely biased our results toward cubozoan-like toxins, which is further exacerbated by the relatively high sequence divergences within the JFT family. However, given that the initial searches within our pipeline used less stringent parameters (see Materials and Methods), we are confident that this is unlikely to be the case. Furthermore, several proteomic studies have identified peptides with close homology to JFTs outside of cubozoans that were not used in our queries, including peptides from a cubozoan (Jaimes-Becerra et al. 2017), scyphozoans (Lassen et al. 2011; Ponce et al. 2015; Jaimes-Becerra et al. 2017; Wang et al. 2018) and, intriguingly, a staurozoan (Jaimes-Becerra and Gacesa et al. 2019), but because these toxins were only identified via mass spectrometry methods, complete coding sequences were unavailable. It is clear that the JFT gene family is more widespread across medusozoans than previously thought, and additional transcriptomic, proteomic, and specifically functional studies will be needed to evaluate the expression of JFTs and their role in various venom repertoires. Additionally, it should be emphasized that medusozoans have varied and complex roles within marine environments that are poorly studied or unknown, and a better understanding of the ecological pressures impacting most species is necessary to better understand how JFTs (and venom function more broadly) influence prey type, prey capture, and predator deterrence across the group.

## Conclusion

Through extensive taxon sampling across Cnidaria, careful yet stringent gene annotation, and by utilizing a phylogenetic approach as well as molecular evolution methods, we provide a more complete view of the distribution of jellyfish toxins (JFTs) and their evolution across Medusozoa. Previous studies that suggested a more taxonomically restricted distribution did not take this multipronged approach and continued species sampling and improved sequencing technologies will certainly lead to additional JFT candidates. Based on our findings, the potency of cubozoan venoms may be the result of a combination of extensive gene duplication and neofunctionalization and/or subfunctionalization. The presence of JFTs in other, less potent species may assist in predation or defense given that JFTs are upregulated in the prey capture–specific zooids/tissues/cells and developing medusae in several species. Although venoms represent a highly complex mixture of proteins and peptides with rampant convergence of venom families, widespread gene duplication, and strong selection pressures, our study shows that using proper annotation and phylogenetic methods can help to uncover complex evolutionary patterns as well as provide a framework to better understand their function.

## Materials and Methods

### Sampled Data

All analyses were conducted using 147 transcriptome assemblies, of which 143 are publicly available. The exceptions are *Sanderia malayensis*, which was assembled using Trinity v2.8.5 (Grabherr, Haas, Yassour et al. 2011; Haas et al. 2013) from publicly available sequence-read archives (SRAs; Kim et al. 2019) (supplementary table S2, Supplementary Material online), and four cerianthid transcriptomes from Klompen et al. (2020), which were built using Trinity v2.2 from publicly available SRAs. In addition, our analysis included the preliminary *H. symbiolongicarpus* genome assembl*y* available at NHGRI (https://research.nhgri.nih.gov/hydractinia/, last accessed April 12, 2019).

### Gene Identification and Annotation

The annotation pipeline described below is illustrated in figure 2. Putative coding regions for each transcriptome were generated using the ORF predication tool TransDecoder v5.5.0 (Haas et al. 2013; https://github.com/TransDecoder, last accessed September 3, 2019) under default settings to create a searchable putative peptide database. Thirteen representative JFTs (table 2) were used as queries to identify putative toxins within this database utilizing two search strategies (e-value cutoff of 0.001): 1) *blastp* search (Blast+ v2.8.1, Camacho et al. 2009) and 2) *hmmsearch* (HMMER v3.1b2, hmmer.org) using a custom hmm-model created with the 13 reference JFTs (available at supplementary file S1, Supplementary Material online). The results from each search were combined and complete ORFs (start and stop codon included) were extracted. CD-HIT (Huang et al. 2010; Fu et al. 2012; http://weizhong-lab.ucsd.edu/cdhit-web-server/, last accessed April 21, 2020) was used to reduce the number of redundant sequences with a cutoff of 95% similarity (-c 0.95). The presence of signal peptides and transmembrane domains were predicted using SignalP v5.0 (Almagro Armenteros et al. 2019) on the SignalP server (http://www.cbs.dtu.dk/services/SignalP/, last accessed April 21, 2020) and TMHMM v2.0 (Krogh et al. 2001) on the TMHMM server (http://www.cbs.dtu.dk/services/TMHMM/, last accessed April 21, 2020), respectively. Only sequences with a signal peptide and either 0 or 1 transmembrane domain were used for further analysis (as in Brinkman et al. 2015). *blastp* searches were conducted against the ToxProt Animal Toxin Annotation (Jungo et al. 2012) and UniProt/SwissProt (The UniProt Consortium 2019) databases (both downloaded in April 2020) to determine if the best match corresponded to a previously described JFT (supplementary table S3, Supplementary Material online). Medusozoan ORFs that passed these filtering criteria and were greater than 300 amino acids in length (based on the sizes of previously described JFTs) were used for phylogenetic analysis.

### Alignment and Phylogenetic Analysis

The 111 putative medusozoan JFTs identified by our annotation pipeline were combined with 13 previously identified JFTs, ten from the reference set with partial sequences excluded and two JFT-like toxins from *H. vulgaris* (Balasubramanian et al. 2012) and one *C. xamachana* sequence (Ames and Klompen et al. 2020) for which there is proteomic evidence, for a total of 124 putative JFTs. This total of 124 putative JFTs were subject to phylogenetic analysis along with six bacterial Cry toxins as outgroups (UniProt accessions: spP16480, spP0A377, spP0A366, spP0A379, spQ06117, and trB3LEP7). Sequences were aligned using MAFFT v7.312 with the L-INS-I algorithm (Katoh and Standley 2013) and ProtTest v3.4.2 was used to determine the best-fit model (Darriba et al. 2011). An ML phylogeny was constructed using RAxML v8.2.12 (Stamatakis 2014) under the GAMMA + I + WAG model with 500 rapid bootstrap replicates (-x 500). The tree was rooted with the six bacterial Cry toxins given these sequences have been previously established as the closest structural matches to JFTs via the I-TASSER server (Brinkman and Burnell 2007; Brinkman et al. 2014). The tree was initially visualized in FigTree v1.4.4 (https://github.com/rambaut/figtree, last accessed November 25, 2018) and the final tree figure edited in Inkscape v1.0beta2 (inkscape.org). Although there is no evidence that anthozoan sequences most similar to JFT’s are expressed as peptides or proteins (within the venom), we also constructed additional phylogenetic trees that included all 124 medusozoan sequences and 15 putative anthozoan JFT sequences that passed our filtering criteria as above (supplementary table S3, Supplementary Material online), both with (supplementary fig. S2, Supplementary Material online) and without (supplementary fig. S3, Supplementary Material online) the bacterial Cry sequences as outgroups.

### Selection Analysis and Molecular Evolution

Because the signal peptide region is under different selection regimes than the mature toxin sequences, the predicted signal peptide region was removed from putative JFTs prior to selection analyses (Sunagar et al. 2014). The overall synonymous to nonsynonymous substitutions (*dN*/*dS*) ratio values were evaluated using Analyze Codon Data analysis (hyphy acd, model = MG94CUSTOMCF3X4) from HyPhy using all sequences for each data set derived from the phylogenetic analyses (i.e., JFT1, JFT2, JFT1b, JFT1c, and all JFTs). Because of the computational power required for these analyses and concerns about the complexity of larger data sets that may compromise specific tests (e.g., BUSTED, aBSREL; Spielman et al. 2019), the total number of candidate toxin ORFs was trimmed by ∼50% while retaining the species diversity in the ML tree. To do so, only one sequence was randomly retained for each species with multiple paralogs within a clade, and the remaining subset of sequences was aligned as above. The random subset selection was performed in triplicate to ensure that specific sequences were not biasing our test results (sequences used in each subset are listed in supplementary table S5, Supplementary Material online). Both *Atolla* sequences were excluded from these analyses because they interfered in our pairwise analyses of major focal clades. Codon alignments were obtained by matching the aligned amino acids to their DNA sequences using *pal2nal* v14 on the online server (Suyama et al. 2006; http://www.bork.embl.de/pal2nal/, last accessed January 8, 2021). An ML phylogeny was constructed for the codon alignment as described above except using the GTR + GAMMA model and 200 rapid bootstrap replicates to ensure similar topology and support as the full ML gene tree. Additionally, all putative JFTs found within the JFT-1 clade from each subset were used in a separate selection analysis. Selection analyses were conducted using HyPhy v2.5.7 (Pond et al. 2005; Kosakovsky Pond et al. 2020), and for all tests both an exploratory search (all branches selected as foreground) and a comparative search (JFT-1 vs. JFT-2; JFT-1b vs. JFT-1c) were conducted. BUSTED v3.0 was used to determine gene-wide episodic positive selection (Murrell et al. 2015), aBSREL v2.1 for branch-specific episodic selection (Smith et al. 2015), and RELAX v3.1 for relaxation or intensification of selection (Wertheim et al. 2015). The resulting significance values for BUSTED (three tests/subset) and RELAX (two tests/subset) have been FDR-corrected using the p.adjust() function in R v3.6.2. (R Core Team 2019); results from aBSREL are default corrected using Holm-Bonferroni. Additionally, two site-specific tests were used on the full data set via the Datamonkey server (Weaver et al 2018; http://www.datamonkey.org, last accessed January 15, 2021): FUBAR v2.2 utilizes a Bayesian approach to detect pervasive positive selection (Murrell et al. 2013) and MEME v2.1.2 uses a mixed-effect model to test for both pervasive and episodic positive selection (Murrell et al. 2012). Tree topologies were modified to be consistent with the ML tree for all tests run through Datamonkey.

### Domain Search and Annotation

Domain structure for all 124 putative JFTs was determined using the MEME-suite v5.1.1 server (Bailey et al. 2015; https://meme-suite.org/meme/, last accessed January 3, 2021) in classic mode using default settings to search for zero or one site per sequence (ZOOPS), except set to search for five possible motifs with a minimum length of 6 and maximum of 200 amino acids. We also compared the JFTs to known domains within Pfam v33.1 (downloaded December 2020; El-Gebali et al. 2019) using *hmmsearch* (HMMER v3.2.1).

### Survey of Differential Expression of JFTs within Tissues and Cells

To determine if JFTs are differentially expressed, we surveyed publicly available RNA-seq studies that identified JFT-like sequences (or included sequences we identified in our own study) as upregulated or downregulated according to the quantification methods described in each study. Because each study utilized a different methodology for quantification and had different expression criteria, these tests are not comparable between studies but can be used to determine trends in JFT expression between similar life stages or tissues. We found data sets for the hydrozoans *H. symbiolognicarpus* (Sanders et al. 2014), *P. carnea* (Sanders and Cartwright 2015a), and *C. hemisphaerica* (Leclère et al. 2019), and for the scyphozoan *A. coerulea* (Gold et al. 2019). We additionally explored the single-cell data set for *Hydra* (Siebert et al. 2019) to determine what cell types JFTs are expressed within.

## Data Availability

All transcriptomic data (assemblies and/or SRA) are publicly available and noted in the supplementary tables S1 and S2, Supplementary Material online. All software used for analyses (including version numbers) are noted in the text. Alignment for the ML trees in figure 3 and supplementary figure S2, Supplementary Material online, are available as supplementary files, Supplementary Material online; alignments used for the selection analyses are available upon request.

## Supplementary Material

Supplementary data are available at *Genome Biology and Evolution* online.

## Supplementary Material

evab081_Supplementary_Data

## References

[evab081-B1] Almagro Armenteros JJ , et al 2019. SignalP 5.0 improves signal peptide predictions using deep neural networks. Nat Biotechnol. 37:420–423 30778233 10.1038/s41587-019-0036-z

[evab081-B2] Ames CL , et al 2020. Cassiosomes are stinging-cell structures in the mucus of the upside-down jellyfish *Cassiopea xamachana*. Commun Biol. 3:67. 32054971 10.1038/s42003-020-0777-8 PMC7018847

[evab081-B3] Anderluh G , Lakey JH. 2008. Disparate proteins use similar architectures to damage membranes. Trends Biochem Sci. 33:482–490. 18778941 10.1016/j.tibs.2008.07.004

[evab081-B4] Anderluh G , Sepčić K, Turk T, Maček P. 2011. Cytolytic proteins from cnidarians: an overview. Acta Chim Slov. 58:724–729 24061121

[evab081-B5] Andreosso A , et al 2018. Structural characterization of predicted helical regions in the *Chironex fleckeri* CfTX-1 toxin. Marine Drugs. 16(6):201. 10.3390/md16060201 PMC6024933 29880743

[evab081-B6] Ashwood LM , Norton RS, Undheim EA, Hurwood DA, Prentis PJ. 2020. Characterizing functional venom profiles of anthozoans and medusozoans within their ecological context. Marine Drugs. 18(4):202. 10.3390/md18040202 PMC7230708 32283847

[evab081-B76165101] Badré S. 2014. Bioactive toxins from stinging jellyfish. Toxicon. 91:114–125. 25286397 10.1016/j.toxicon.2014.09.010

[evab081-B7] Bailey TL , Johnson J, Grant CE, Noble WS. 2015. The MEME suite. Nucleic Acids Res. 43(W1):W39–W49. 25953851 10.1093/nar/gkv416 PMC4489269

[evab081-B8] Balasubramanian PG , et al 2012. Proteome of Hydra nematocyst. J Biol Chem. 28(13):79672–9681. 10.1074/jbc.M111.328203 PMC3323026 22291027

[evab081-B9] Bentlage B , et al 2010. Evolution of box jellyfish (Cnidaria: cubozoa), a group of highly toxic invertebrates. Proc Biol Sci. 277(1680):493–501. 19923131 10.1098/rspb.2009.1707 PMC2842657

[evab081-B10] Brinkman DL , Burnell JN. 2007. Identification, cloning and sequencing of two major venom proteins from the box jellyfish, *Chironex fleckeri*. Toxicon 50(6):850–860. 17688901 10.1016/j.toxicon.2007.06.016

[evab081-B11] Brinkman DL , Burnell JN. 2008. Partial purification of cytolytic venom proteins from the box jellyfish, *Chironex fleckeri*. Toxicon 51(5):853–863. 18243272 10.1016/j.toxicon.2007.12.017

[evab081-B12] Brinkman DL , Burnell JN. 2009. Biochemical and molecular characterization of cubozoan protein toxins. Toxicon 54(8):1162–1173. 19232527 10.1016/j.toxicon.2009.02.006

[evab081-B13] Brinkman DL , et al 2014. *Chironex fleckeri* (box jellyfish) venom proteins: expansion of a cnidarian toxin family that elicits variable cytolytic and cardiovascular effects. J Biol Chem. 289(8):4798–4812. 24403082 10.1074/jbc.M113.534149 PMC3931041

[evab081-B14] Brinkman DL , et al 2015. Transcriptome and venom proteome of the box jellyfish *Chironex fleckeri*. BMC Genomics 16:407. 26014501 10.1186/s12864-015-1568-3 PMC4445812

[evab081-B15] Camacho C , et al 2009. BLAST+: architecture and applications. BMC Bioinformatics 10:421. 20003500 10.1186/1471-2105-10-421 PMC2803857

[evab081-B16] Chang ES et al 2015. Genomic insights into the evolutionary origin of Myxozoa within Cnidaria. Proc Natl Acad Sci U S A. 112(48):14912–14917. 26627241 10.1073/pnas.1511468112 PMC4672818

[evab081-B17] Chung JJ , Ratnapala LA, Cooke IM, Yanagihara AA. 2001. Partial purification and characterization of a hemolysin (*CAH1*) from Hawaiian box jellyfish (Carybdea alata) venom. Toxicon. 39(7):981–990. 10.1016/S0041-0101(00)00237-3 11223087

[evab081-B18] Columbus-Shenkar YY , et al 2018. Dynamics of venom composition across a complex life cycle. eLife 7:e35014. 29424690 10.7554/eLife.35014 PMC5832418

[evab081-B19] Courtney R , Sachlikidis N, Jones R, Seymour J. 2015. Prey capture ecology of the cubozoan *Carukia barnesi*. PLoS One 10(5):e0124256. 25970583 10.1371/journal.pone.0124256 PMC4429964

[evab081-B20] Darriba D , Taboada GL, Doallo R, Posada D. 2011. ProtTest 3: fast selection of best-fit models of protein evolution. Bioinformatics 27(8):1164–1165. 21335321 10.1093/bioinformatics/btr088 PMC5215816

[evab081-B21] Dutertre S , et al 2014. Evolution of separate predation-and defence-evoked venoms in carnivorous cone snails. Nat Commun. 5:3521. 24662800 10.1038/ncomms4521 PMC3973120

[evab081-B22] El-Gebali S et al 2019. The Pfam protein families database in 2019. Nucleic Acids Res. 47(D1):D427–D432. 30357350 10.1093/nar/gky995 PMC6324024

[evab081-B23] Feng J , et al 2010. Partial characterization of the hemolytic activity of the nematocyst venom from the jellyfish *Cyanea nozakii* Kishinouye. Toxicol In Vitro. 24(6):1750–1756. 20156548 10.1016/j.tiv.2010.02.010

[evab081-B24] Fenner PJ , Williamson JA. 1996. Worldwide deaths and severe envenomation from jellyfish stings. Med J. Aust. 165(11–12):658–661. 8985452 10.5694/j.1326-5377.1996.tb138679.x

[evab081-B25] Fu L , Niu B, Zhu Z, Wu S, Li W. 2012. CD-HIT: accelerated for clustering the next-generation sequencing data. Bioinformatics 28(23):3150–3152. 23060610 10.1093/bioinformatics/bts565 PMC3516142

[evab081-B26] Gacesa R , et al 2015. Gene duplications are extensive and contribute significantly to the toxic proteome of nematocysts isolated from *Acropora digitifera* (Cnidaria: anthozoa: scleractinia) BMC Genomics 16:774. 26464356 10.1186/s12864-015-1976-4 PMC4604070

[evab081-B27] Gold DA , et al 2019. The genome of the jellyfish *Aurelia* and the evolution of animal complexity. Nat Ecol Evol. 3(1):96–104. 30510179 10.1038/s41559-018-0719-8

[evab081-B28] Grabherr MG , et al 2011. Full-length transcriptome assembly from RNA-Seq data without a reference genome. Nat Biotechnol. 29(7):644–652. 21572440 10.1038/nbt.1883 PMC3571712

[evab081-B29] Haas BJ , et al 2013. *De novo* transcript sequence reconstruction from RNA-seq using the Trinity platform for reference generation and analysis. Nat Protoc. 8:1494–1512. 23845962 10.1038/nprot.2013.084 PMC3875132

[evab081-B30] Hamner W , Jones M, Hamner P. 1995. Swimming, feeding, circulation and vision in the Australian box jellyfish, *Chironex fleckeri* (Cnidaria: cubozoa). Mar Freshwater Res. 46(7):985.

[evab081-B31] Hessinger DA. 1988. Nematocysts venoms and toxins. In: Hessinger DA, Lenhoff HM, editors. The biology of nematocysts. San Diego (CA): Academic Press Inc. p. 333–369.

[evab081-B32] Herzig V , et al 2020. Animal toxins—nature’s evolutionary-refined toolkit for basic research and drug discovery Biochem Pharmacol. 181:114096. 32535105 10.1016/j.bcp.2020.114096 PMC7290223

[evab081-B33] Huang Y , Niu B, Gao Y, Fu L, Li W. 2010. CD-HIT suite: a web server for clustering and comparing biological sequences. Bioinformatics. 26(5): 680–682. 20053844 10.1093/bioinformatics/btq003 PMC2828112

[evab081-B34] Jaimes-Becerra A , et al 2017. Comparative proteomics reveals recruitment patterns of some protein families in the venoms of Cnidaria. Toxicon 137:19–26. 28711466 10.1016/j.toxicon.2017.07.012

[evab081-B35] Jaimes-Becerra A , et al 2019. “Beyond primary sequence”—proteomic data reveal complex toxins in cnidarian venoms. Integr Comp Biol. 59(4):777–785. 31225595 10.1093/icb/icz106

[evab081-B36] Jouiaei M , Sunagar K, et al 2015. Evolution of an ancient venom: recognition of a novel family of cnidarian toxins and the common evolutionary origin of sodium and potassium neurotoxins in sea anemone. Mol Biol Evol. 32(6):1598–1610. 25757852 10.1093/molbev/msv050

[evab081-B37] Jouiaei M , Yanagihara AA, et al 2015. Ancient venom systems: a review on Cnidaria toxins. Toxins 7(6):2251–2271. 26094698 10.3390/toxins7062251 PMC4488701

[evab081-B38] Jungo F , Bougueleret L, Xenarios I, Poux S. 2012. The UniProtKB/Swiss-Prot Tox-Prot program: a central hub of integrated venom protein data. Toxicon 60(4):551–557. 22465017 10.1016/j.toxicon.2012.03.010 PMC3393831

[evab081-B39] Katoh K , Standley DM. 2013. MAFFT multiple sequence alignment software version 7: improvements in performance and usability. Mol Biol Evol.. 30(4):772–780. 23329690 10.1093/molbev/mst010 PMC3603318

[evab081-B40] Kass-Simon GS , Scappaticci AA. 2002. The behavioral and developmental physiology of nematocysts. Can J Zool. 80(10):177–294.

[evab081-B41] Kawabata T , et al 2013. Evaluation of the bioactivities of water-soluble extracts from twelve deep-sea jellyfish species. Fish Sci. 79(3):487–494.

[evab081-B42] Kayal E , et al 2018. Phylogenomics provides a robust topology of the major cnidarian lineages and insights on the origins of key organismal traits. BMC Evol Biol. 48:68.

[evab081-B43] Killi N , et al 2020. Nematocyst types and venom effects of *Aurelia aurita* and *Velella velella* from the Mediterranean sea. Toxicon 175:57–63. 32056696 10.1016/j.toxicon.2019.12.155

[evab081-B44] Kim HM , et al 2019. The genome of the giant Nomura’s jellyfish sheds light on the early evolution of active predation. BMC Biol. 172(1):8. 10.1186/s12915-019-0643-7 PMC6441219 30925871

[evab081-B45] Klompen AML , Macrander J, Reitzel AM, Stampar SN. 2020. Transcriptomic analysis of four cerianthid (Cnidaria, Ceriantharia) venoms. Mar Drugs. 18(8):413. 10.3390/md18080413 PMC7460484 32764303

[evab081-B46] Kosakovsky Pond SL , et al 2020. HyPhy 2.5: a customizable platform for evolutionary hypothesis testing using phylogenies Mol Biol Evol. 37(1):295–299. 31504749 10.1093/molbev/msz197 PMC8204705

[evab081-B47] Krogh A , Larsson B, von Heijne G, Sonnhammer ELL. 2001. Predicting transmembrane protein topology with a hidden Markov model: application to complete. J Mol Biol. 305(3): 567–580. 11152613 10.1006/jmbi.2000.4315

[evab081-B48] Lassen S , Helmholz H, Ruhnau C, Prange A. 2011. A novel proteinaceous cytotoxin from the northern scyphozoa *Cyanea capillata* (L.) with structural homology to cubozoan haemolysins. Toxicon 57(5):721–729. 21333668 10.1016/j.toxicon.2011.02.004

[evab081-B49] Lawley JW , et al 2016. Box jellyfish *Alatina alata* has a circumtropical distribution. Biol Bull. 231(2):152–169. 27820907 10.1086/690095 PMC5599302

[evab081-B50] Leclère L , et al 2019. The genome of the jellyfish *Clytia hemisphaerica* and the evolution of the cnidarian life-cycle. Nat Ecol Evol. 3(5):801–810. 30858591 10.1038/s41559-019-0833-2

[evab081-B51] Lewis C , Bentlage B. 2009. Clarifying the identity of the Japanese Habu-kurage, *Chironex yamaguchii*, sp. nov. (Cnidaria: cubozoa: chirodropida). Zootaxa 2030:59–65.

[evab081-B52] Lewis Ames C , Macrander J. 2016. Evidence for an alternative mechanism of toxin production in the box jellyfish *Alatina alata*. Integr Comp Biol. 56(5):973–988. 27880678 10.1093/icb/icw113

[evab081-B53] Lewis Ames C , Ryan JF, Bely AE, Cartwright P, Collins AG. 2016. A new transcriptome and transcriptome profiling of adult and larval tissue in the box jellyfish *Alatina alata*: an emerging model for studying venom, vision and sex. BMC Genomics 17:650. 27535656 10.1186/s12864-016-2944-3 PMC4989536

[evab081-B54] Li R , et al 2014. Jellyfish venomics and venom gland transcriptomics analysis of *Stomolophus meleagris* to reveal the toxins associated with sting. J Proteomics. 106:17–29. 24747124 10.1016/j.jprot.2014.04.011

[evab081-B55] Li R , Yu H, Li T, Li P. 2020. Comprehensive proteome reveals the key lethal toxins in the venom of jellyfish *Nemopilema nomurai*. J Proteome Res.19(6):2491–2500. 32374608 10.1021/acs.jproteome.0c00277

[evab081-B56] Liu G , et al 2015. Global transcriptome analysis of the tentacle of the jellyfish *Cyanea capillata* using deep sequencing and expressed sequence tags: insight into the toxin- and degenerative disease-related transcripts. PLoS One 10(11):e0142680. 26551022 10.1371/journal.pone.0142680 PMC4638339

[evab081-B57] Mariottini G , Pane L. 2013. Cytotoxic and cytolytic cnidarian venoms. A review on health implications and possible therapeutic applications. Toxins 6(1):108–151. 24379089 10.3390/toxins6010108 PMC3920253

[evab081-B58] Murrell B , et al 2012. Detecting individual sites subject to episodic diversifying selection. PLoS Genet. 8(7):e1002764. 22807683 10.1371/journal.pgen.1002764 PMC3395634

[evab081-B59] Murrell B , et al 2013. FUBAR: a fast, unconstrained Bayesian approximation for inferring selection. Mol Biol Evol. 30(5):1196–1205. 23420840 10.1093/molbev/mst030 PMC3670733

[evab081-B60] Murrell B , et al 2015. Gene-wide identification of episodic selection. Mol Biol Evol. 32(5):1365–1371. 25701167 10.1093/molbev/msv035 PMC4408417

[evab081-B61] Nagai H , Takuwa-Kuroda K, et al 2000. Novel proteinaceous toxins from the box jellyfish (sea wasp) *Carybdea rastoni*. Biochem Biophys Res Commun. 275(2):582–588. 10964707 10.1006/bbrc.2000.3353

[evab081-B62] Nagai H , Takuwa-Kuroda K, Nakao M, et al 2000. Isolation and characterization of a novel protein toxin from the Hawaiian box jellyfish (sea wasp) *Carybdea alata*. Biochem Biophys Res Commun. 275(2):589–594. 10964708 10.1006/bbrc.2000.3352

[evab081-B63] Nagai H , et al 2002. A novel protein toxin from the deadly box jellyfish (sea wasp, Habu-kurage) *Chiropsalmus quadrigatus*. Biosci Biotechnol Biochem. 66(1):97–102. 11866126 10.1271/bbb.66.97

[evab081-B64] Nagai H. 2003. Recent progress in jellyfish toxin study. J Health Sci. 49(5):337–340.

[evab081-B65] Orellana ER , Collins AG. 2011. First report of the box jellyfish *Tripedalia cystophora* (Cubozoa: tripedaliidae) in the continental USA, from Lake Wyman, Boca Raton, Florida. Mar Biodivers Rec. 4:e54.

[evab081-B66] Peraro MD , van der Goot FG. 2016. Pore-forming toxins: ancient, but never really out of fashion. Nat Rev Microbiol. 14(2):77–92. 26639780 10.1038/nrmicro.2015.3

[evab081-B67] Podobnik M , Anderluh G. 2017. Pore-forming toxins in Cnidaria. Semin Cell Dev Biol. 72:133–141. 28751252 10.1016/j.semcdb.2017.07.026

[evab081-B68] Ponce D , Brinkman D, Potriquet J, Mulvenna J. 2016. Tentacle transcriptome and venom proteome of the pacific sea nettle, *Chrysaora fuscescens* (Cnidaria: scyphozoa). Toxins 8(4):102. 27058558 10.3390/toxins8040102 PMC4848628

[evab081-B69] Ponce D , Brinkman DL, Luna-Ramírez K, Wright CE, Dorantes-Aranda JJ. 2015. Comparative study of the toxic effects of *Chrysaora quinquecirrha* (Cnidaria: scyphozoa) and *Chironex fleckeri* (Cnidaria: cubozoa) venoms using cell-based assays. Toxicon 106:57–67. 26385314 10.1016/j.toxicon.2015.09.014

[evab081-B70] Pond SLK , Frost SDW, Muse SV. 2005. HyPhy: hypothesis testing using phylogenies. Bioinformatics. 21:676–679. 15509596 10.1093/bioinformatics/bti079

[evab081-B71] R Core Team. 2019. A language and environment for statistical computing. In: R foundation for statistical computing. Vienna (Austria). https://www.R-project.org/. Date accessed January 13, 2020.

[evab081-B72] Rachamim T , et al 2015. The dynamically evolving nematocyst content of an anthozoan, a scyphozoan, and a hydrozoan. Mol Biol Evol. 32(3):740–753. 25518955 10.1093/molbev/msu335

[evab081-B73] Radwan FFY , et al 2001. A comparison of the toxinological characteristics of two *Cassiopea* and *Aurelia* species. Toxicon 39(2–3):245–257. 10978742 10.1016/s0041-0101(00)00121-5

[evab081-B74] Sachkova MY , et al 2019. The birth and death of toxins with distinct functions: a case study in the sea anemone *Nematostella*. Mol Biol Evol. 36(9):2001–2012. 31134275 10.1093/molbev/msz132

[evab081-B75] Sanders SM , Shcheglovitova M, Cartwright P. 2014. Differential gene expression between functionally specialized polyps of the colonial hydrozoan *Hydractinia symbiolongicarpus* (Phylum Cnidaria). BMC Genomics 15:406. 24884766 10.1186/1471-2164-15-406 PMC4072882

[evab081-B76] Sanders SM , Cartwright P. 2015a. Patterns of Wnt signaling in the life cycle of *Podocoryna carnea* and its implications for medusae evolution in Hydrozoa (Cnidaria): Wnt signaling and medusae evolution. Evol Dev. 17(6):325–336. 26487183 10.1111/ede.12165

[evab081-B77] Sanders SM , Cartwright P. 2015b. Interspecific differential expression analysis of RNA-Seq data yields insight into life cycle variation in hydractiniid hydrozoans. Genome Biol Evol. 7(8):2417–2431. 26251524 10.1093/gbe/evv153 PMC4558869

[evab081-B78] Siebert S , et al 2019. Stem cell differentiation trajectories in *Hydra* resolved at single-cell resolution. Science 365(6451):eaav9314. 31346039 10.1126/science.aav9314 PMC7104783

[evab081-B79] Schendel V , Rash LD, Jenner RA, Undheim EA. 2019. The diversity of venom: the importance of behavior and venom system morphology in understanding its ecology and evolution. Toxins 11:666. 10.3390/toxins11110666 PMC6891279 31739590

[evab081-B80] Sher D , et al 2005. Toxic polypeptides of the *Hydra*: a bioinformatic approach to cnidarian allomones. Toxicon 45(7):865–879. 15904682 10.1016/j.toxicon.2005.02.004

[evab081-B81] Smith MD , et al 2015. Less is more: an adaptive branch-site random effects model for efficient detection of episodic diversifying selection. Mol Evol Biol. 32(5):1342–1353. 10.1093/molbev/msv022 PMC4408413 25697341

[evab081-B82] Spielman SJ , et al 2019. Evolution of viral genomes: interplay between selection, recombination, and other forces. In: Anisimova M, editor. Evolutionary genomics: statistical and computational methods. Methods in molecular biology. Vol. 1910. New York (NY): Humana. p. 427–468. 10.1007/978-1-4939-9074-0_14 31278673

[evab081-B83] Stamatakis A. 2014. RAxML version 8: a tool for phylogenetic analysis and post-analysis of large phylogenies. Bioinformatics 30(9):1312–1313. 24451623 10.1093/bioinformatics/btu033 PMC3998144

[evab081-B84] Stewart SE. 1996. Field behavior of *Tripedalia cystophora* (class Cubozoa) Mar Freshw Behav Physiol. 27(2–3):175–188.

[evab081-B85] Stoner EW , et al 2014. Modification of a seagrass community by benthic jellyfish blooms and nutrient enrichment. J Exp Mar Biol Ecol. 461:185–192.

[evab081-B86] Sunagar K , et al 2014. Deadly innovations: unraveling the molecular evolution of animal venoms. In: Gopalakrishnakone P, Calvete J, editors. Venom genomics and proteomics. Dordrecht (Netherlands): Springer. p. 1–23.

[evab081-B87] Sunagar K , Moran Y. 2015. The rise and fall of an evolutionary innovation: contrasting strategies of venom evolution in ancient and young animals. PLoS Genet. 11(10):e1005596. 26492532 10.1371/journal.pgen.1005596 PMC4619613

[evab081-B88] Surm JM , et al 2019. A process of convergent amplification and tissue‐specific expression dominates the evolution of toxin and toxin‐like genes in sea anemones. Mol Ecol. 28(9):2272–2289. 30913335 10.1111/mec.15084

[evab081-B89] Surm JM , Moran Y. 2021. Insights into how development and life-history dynamics shape the evolution of venom. EvoDevo 12(1). 10.1186/s13227-020-00171-w PMC7791878 33413660

[evab081-B90] Suyama M , Torrents D, Bork P. 2006. PAL2NAL: robust conversion of protein sequence alignments into the corresponding codon alignments. Nucleic Acids Res. 34(Web Server Issue):W609–W612. 16845082 10.1093/nar/gkl315 PMC1538804

[evab081-B91] The UniProt Consortium. 2019. UniProt: a worldwide hub of protein knowledge. Nucleic Acids Res. 47:D506–D515. 30395287 10.1093/nar/gky1049 PMC6323992

[evab081-B92] Tamkun MM , Hessinger DA. 1981. Isolation and partial characterization of a hemolytic and toxic protein from the nematocyst venom of the Portuguese Man-of-War, *Physalia physalis*. Biochim Biophys Acta. 667(1):87–98. 6111356 10.1016/0005-2795(81)90069-6

[evab081-B93] Toshino S , Miyake H, Shibata H. 2015. *Meteorona kishinouyei*, a new family, genus and species (Cnidaria, Cubozoa, Chirodropida) from Japanese Waters ZooKeys. 50: 31. 10.3897/zookeys.503.9047 PMC4440269 26019668

[evab081-B94] Turk T , Kem WR. 2009. The phylum Cnidaria and investigations of its toxins and venoms until 1990. Toxicon 54(8):1031–1037. 19576920 10.1016/j.toxicon.2009.06.031

[evab081-B95] Underwood AH , Seymour JE. 2007. Venom ontogeny, diet and morphology in *Carukia barnesi*, a species of Australian box jellyfish that causes Irukandji syndrome. Toxicon 49(8):1073–1082. 17395227 10.1016/j.toxicon.2007.01.014

[evab081-B96] Wang C , et al 2018. Unique diversity of sting-related toxins based on transcriptomic and proteomic analysis of the jellyfish *Cyanea capillata* and *Nemopilema nomurai* (Cnidaria: scyphozoa). J Proteome Res. doi:10.1021/acs.jproteome.8b00735. 30481029

[evab081-B97] Weaver S et al 2018. Datamonkey 2.0: a modern web application for characterizing selective and other evolutionary processes. Mol Biol Evol. 35(3):773–777. 29301006 10.1093/molbev/msx335 PMC5850112

[evab081-B98] Wertheim JO , Murrell B, Smith MD, Kosakovsky Pond SL, Scheffler K. 2015. RELAX: detecting relaxed selection in a phylogenetic framework. Mol Biol Evol. 32(3):820–832. 25540451 10.1093/molbev/msu400 PMC4327161

[evab081-B99] WoRMS Editorial Board. 2021. World Register of Marine Species. Available from: http://www.marinespecies.org at VLIZ. Date accessed January 26, 2021.

[evab081-B100] Yanagihara AA , Shohet RV. 2012. Cubozoan venom-induced cardiovascular collapse is caused by hyperkalemia and prevented by zinc gluconate in mice. PLoS One 7(12):e51368. 23251508 10.1371/journal.pone.0051368 PMC3520902

[evab081-B101] Yanagihara AA , Wilcox C, Smith J, Surrett GW. 2016. Cubozoan envenomations: clinical features, pathophysiology and management. In: Goffredo S, Dubinsky Z, editors. The Cnidaria, past, present and future. Cham: Springer International Publishing. p. 637–652.

[evab081-B102] Zapata F , et al 2015. Phylogenomic analyses support traditional relationships within Cnidaria PLoS One 10(10):e0139068. 26465609 10.1371/journal.pone.0139068 PMC4605497

