# Supplementary material for: Phylogenetic and Selection Analysis of an Expanded Family of Putatively Pore-Forming Jellyfish Toxins (Cnidaria: Medusozoa)
Source: Genome Biol Evol. 2021 Apr 23;13(6):evab081. doi: 10.1093/gbe/evab081 (PMC8214413; doi:10.1093/gbe/evab081)

A

Transmembrane spanning region 1

```
CfTX-1     64 QSGDPAKIASGCLDILVGISSVLKD------FAKFSPIFSILSLVVGLFSGTKAEESVGSVVKKAVQEQS 127
CfTX-2     64 QSGDPASIASGCLDILVGISSVLKD------FAKFSPVFSILSLVVGLFSGTKAEESVSSVVTKAIQEQS 127
CqTX-A     64 QSGDPAKIASGCLDILVGISSVLKD------FAKFSPIFSILSMVVGLFSGTKAEESVGSVVKKVVQEQS 127
CrTX-1     65 NSGDATKIISGCLDIVAGIATTFGGPVGMGIGAVASFVSSILSL----FTGSSAKNSVAAVIDRALSKHR 130
CaTX-1     71 KSGDPFDVASGCLDIIASVATTFGGPYGIAIGAVASLISSILSL----FSGNSMGSAIKQVIDDAFKKYR 136
```

MEME - Motif 2

B

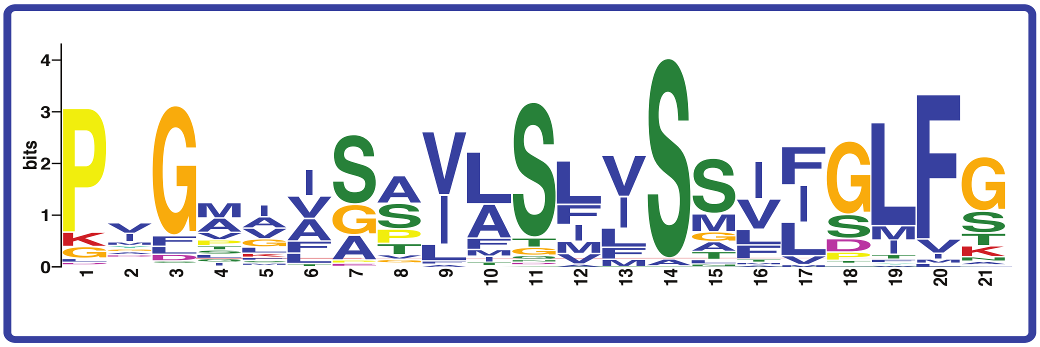

Supplement: evab081_Supplementary_Data [file evab081_supplementary_data.zip › SuppFigureS1.pdf]
