# Supplementary material for: Phylogenetic and Selection Analysis of an Expanded Family of Putatively Pore-Forming Jellyfish Toxins (Cnidaria: Medusozoa)
Source: Genome Biol Evol. 2021 Apr 23;13(6):evab081. doi: 10.1093/gbe/evab081 (PMC8214413; doi:10.1093/gbe/evab081)

A

		Branches under episodic selection	p-value (Holm-Bonferroni corrected)
**SUBSET**	JFT-1b	Node2	0.00000
	JFT-1c	/	/
	TOTAL	Node2	0.00000
**FULL**	JFT-1	/	/
	JFT-2	Node30	0.00000
		Node26	0.04181
		GHAS01089849.1 *Aurelia aurita* (Kuji)	0.00010
		GHAG01088146.1 *Aurelia aurita* (Baltic)	0.01038
	TOTAL	Node30	0.00000
		GHAS01089849.1 *Aurelia aurita* (Kuji)	0.02168
		GHAG01088146.1 *Aurelia aurita* (Baltic)	0.00000

B

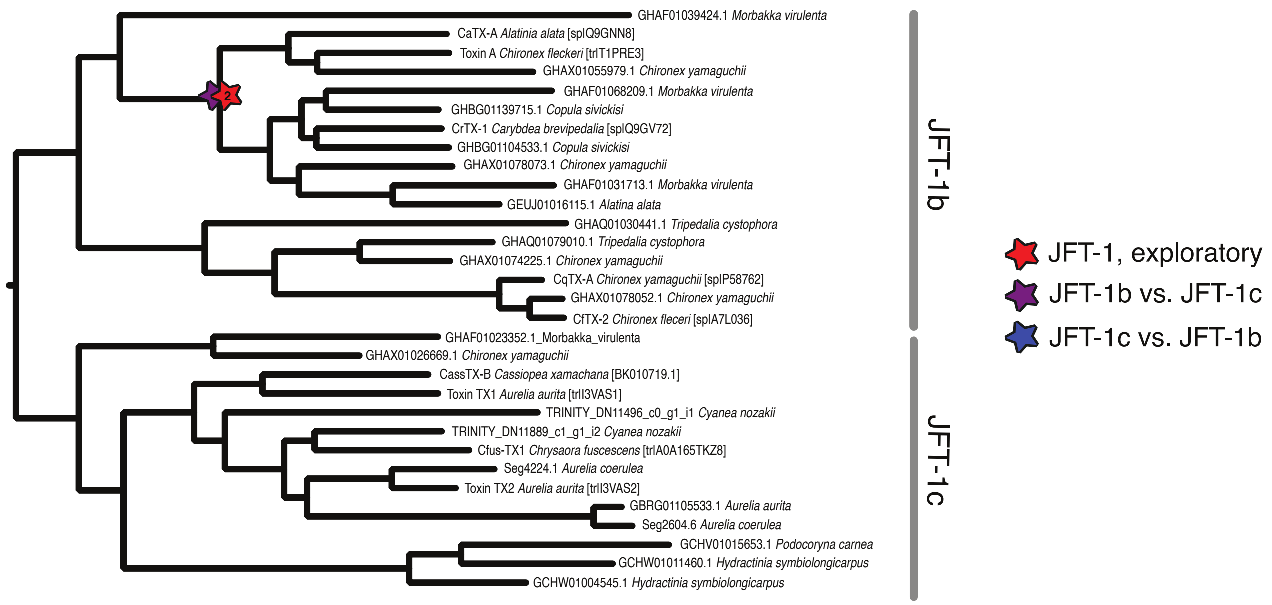

C

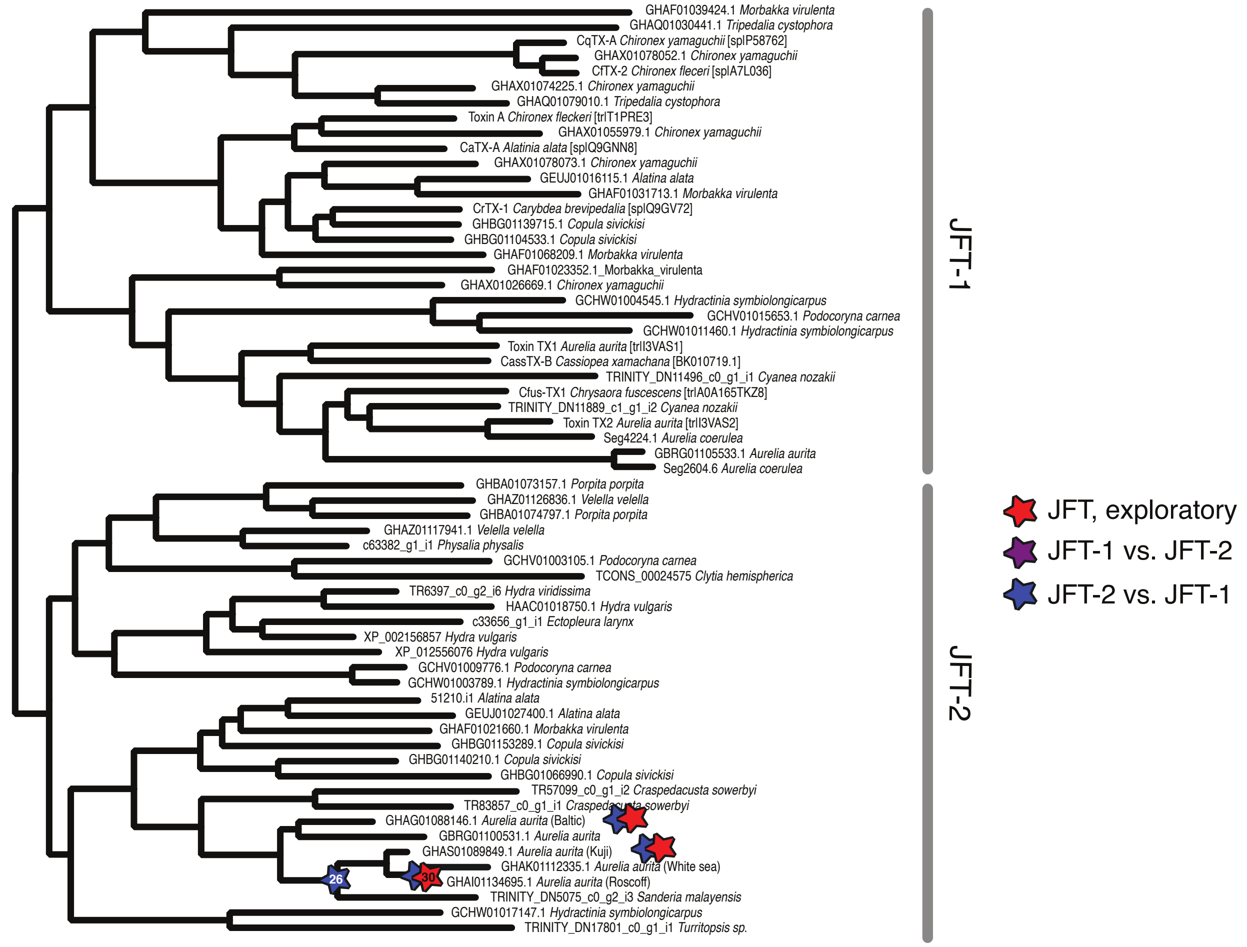

Supplement: evab081_Supplementary_Data [file evab081_supplementary_data.zip › SuppFigureS4.pdf]
