# Supplementary material for: Phylogenetic and Selection Analysis of an Expanded Family of Putatively Pore-Forming Jellyfish Toxins (Cnidaria: Medusozoa)
Source: Genome Biol Evol. 2021 Apr 23;13(6):evab081. doi: 10.1093/gbe/evab081 (PMC8214413; doi:10.1093/gbe/evab081)

A

		Branches under episodic selection	p-value (Holm-Bonferroni corrected)
**SUBSET**	JFT-1b	Node56	0.00060
		Node28	0.00248
	JFT-1c	/	/
	TOTAL	Node56	0.00099
		Node28	0.00413
**FULL**	JFT-1	Node6	0.00196
	JFT-2	Seg1153.8 (*Aurelia coerulea*)	0.00000
	TOTAL	Seg1153.8 (*Aurelia coerulea*)	0.00000
		Node6	0.00392

B

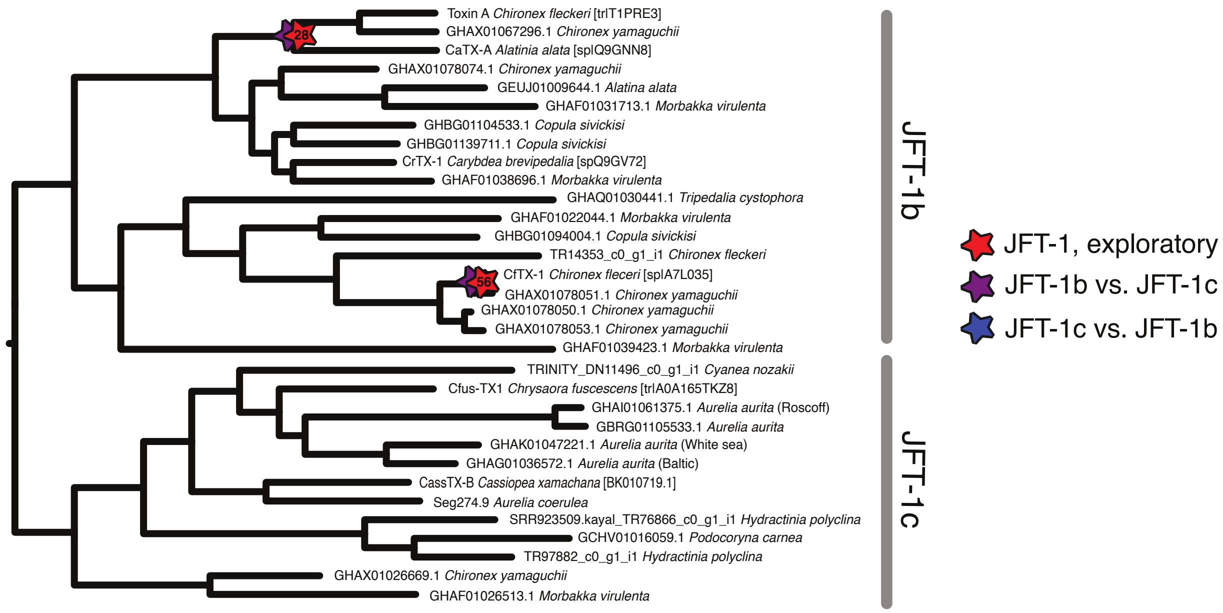

C

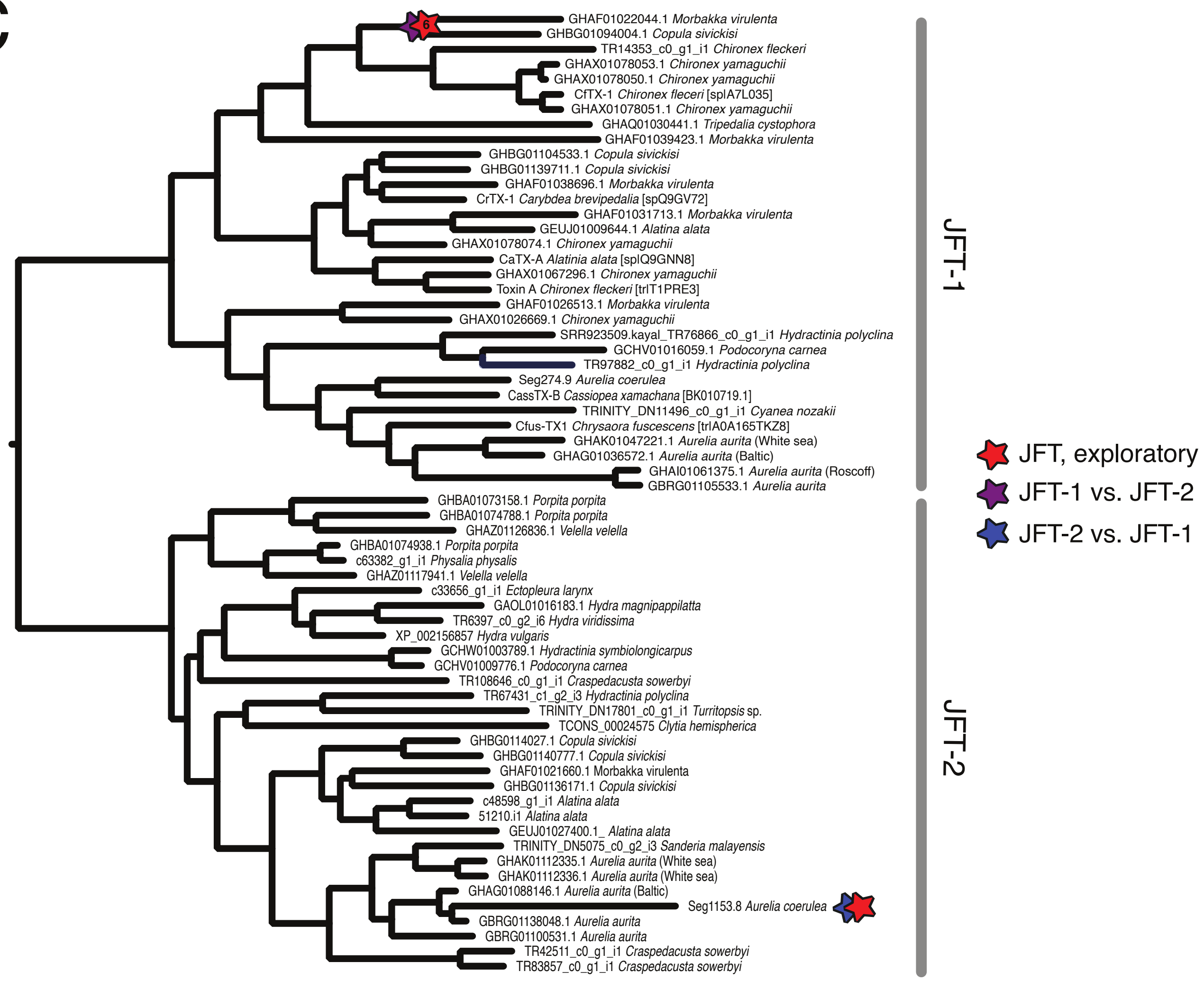

Supplement: evab081_Supplementary_Data [file evab081_supplementary_data.zip › SuppFigureS5.pdf]
