# Supplementary material for: Phylogenetic and Selection Analysis of an Expanded Family of Putatively Pore-Forming Jellyfish Toxins (Cnidaria: Medusozoa)
Source: Genome Biol Evol. 2021 Apr 23;13(6):evab081. doi: 10.1093/gbe/evab081 (PMC8214413; doi:10.1093/gbe/evab081)

A

		Branches under episodic selection	p-value (Holm-Bonferroni corrected)
**SUBSET**	JFT-1b	CqTXA *Chironex yamaguchii* [spP58762]	0.00227
	JFT-1c	Node50	0.00026
	TOTAL	Node50	0.00064
**FULL**	JFT-1	CqTXA *Chironex yamaguchii* [spP58762]	0.00378
		Node63	0.00000
		Node83	0.01087
	JFT-2	CqTXA *Chironex yamaguchii* [spP58762]	0.00013
		GHAI01134692.1 *Aurelia aurita* (Roscoff)	0.00013
		GEVZ01006697.1 *Hydra vulgaris*	0.00129
		GHAK01112335.1 *Aurelia aurita* (White sea)	0.00322
	TOTAL	Node63	0.00000
		Node83	0.02067
		CqTXA *Chironex yamaguchii* [spP58762]	0.00026
		GHAI01134692.1 *Aurelia aurita* (Roscoff)	0.00026
		GEVZ01006697.1 *Hydra vulgaris*	0.00268
		GHAK01112335.1 *Aurelia aurita* (White sea)	0.00673

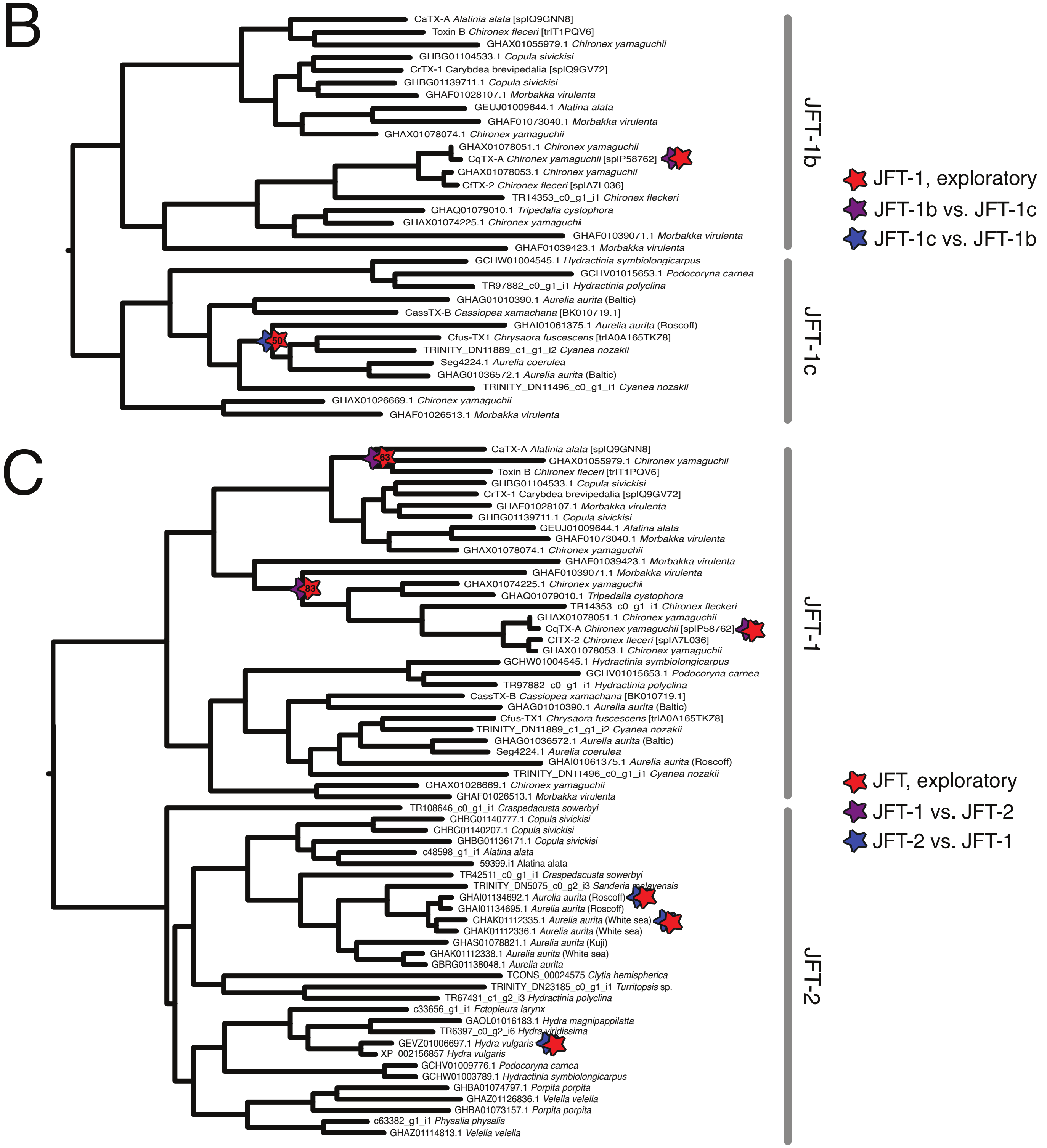

Supplement: evab081_Supplementary_Data [file evab081_supplementary_data.zip › SuppFigureS6.pdf]
