# Supplementary material for: Phylogenetic and Selection Analysis of an Expanded Family of Putatively Pore-Forming Jellyfish Toxins (Cnidaria: Medusozoa)
Source: Genome Biol Evol. 2021 Apr 23;13(6):evab081. doi: 10.1093/gbe/evab081 (PMC8214413; doi:10.1093/gbe/evab081)
Supplement: evab081_Supplementary_Data [file evab081_supplementary_data.zip › Supplementary Materials.R2.docx]

**Supplementary Information**

**Supplementary Files**

**Supplementary File S1: Custom HMM-model created using 13 JFT reference sequences.** (See File_S1.txt file.)

**Supplementary File S2: Multiple sequence alignment of medusozoan JFTs with Cry bacterial toxins used for maximum likelihood reconstruction.** Final tree shown in Figure 3. (See File_S2.fa file.)

**Supplementary File S3: Multiple sequence alignment of medusozoan and anthozoan JFTs with Cry bacterial toxins used for maximum likelihood reconstruction.** Final tree shown in in supplementary figure S2. (See File_S3.fa file.)

**Supplementary File S4: Nucleotide sequences for 124 medusozoan JFTs sequences.** (See File_S4.fa file.)

**Supplementary Tables**

**Table S1: List of sequences and peptide references that have been previously reported as JFTs or JFT-like genes.** A number followed by an * indicates that multiple paralogs were identified within that species. Red indicates genes that were identified in the present study and used in the phylogenetic analysis. T = transcriptome, G = genome, P = peptide/protein. (See Table_S1.xlxs file.)

**Table S2:** **Transcriptomes assemblies and SRA availability used for this study.** (See Table_S2.xlxs file.)

**Table S3:** **Annotation table for total putative JFTs.** Putative JFTs and corresponding annotations (“JFT_unfiltered,” sheet 1) and the filtered dataset of JFTs used for the final phylogenetic tree (“JFTs_on_tree,” sheet 2) are shown. Gray colored cells on the unfiltered dataset indicates anthozoan JFT-like sequences that passed the filtering criteria and were used in subsequence supplementary trees (Supplemental Figures S2, S3). The filtered dataset is colored as such: red for cubozoans, yellow for scyphozoans, and blue for hydrozoans. (See Table_S3.xlxs file.)

**Table S4: Results from exploratory domain identification for putative medusozoan JFTs.** The top three results from a hmmsearch against the Pfam database are shown for the filtered medusozoan dataset (“Pfam_results,” sheet 1). The output of MEME-suite for motif 2 is shown, including corresponding e-values and sequence information (“MEME_motif2,” sheet 2). Of note, Seg1153.8 (*Aurelia coerulea*) and GHAF01039071.1 (*Morbakka virulenta*) did not have a significant match (p-value < 0.00001) to this motif. (See Table_S4.xlxs file.)

**Table S5: Additional outcomes from HyPhy for site-specific tests, RELAX, as well as lists of subset data used in gene- and branch-specific analyses.** Output from FUBAR analysis for all putative medusozoan JFTs (124 sequences) (“FUBAR_JFT_total,” sheet 1). Output from MEME analysis for medusozoan JFTs (124 sequences) (“MEME_JFT_total,” sheet 2). Both FUBAR and MEME were used via the Datamonkey server (accessed January 2021, datamonkey.org; Weaver et al. 2018), and header descriptors are modified from output files and descriptions in Spielman et al. (2019). Q-values (FDR-corrected p-values) were calculated for MEME results using p.adjust() in R. 3) Results of RELAX analysis for each of the trimmed medusozoans subsets. (*) indicates tests that were run using the Datamonkey server with trees adjusted to match the maximum likelihood tree (accessed January 2021, datamonkey.org) (“RELAX_results,” sheet 3). 4) Sequences used in each subset for BUSTED, RELAX, and aBSREL tests (“Subsets,” sheet 4) (exception of Seg1153.8 (*Aurelia coerulea*) that was trimmed for the RELAX test of JFT-2 versus JFT-1 in subset 2). (See Table_S5.xlxs file.)

**Supplementary Figures**

**Figure S1: Putative transmembrane spanning region motif of JFT sequences.** A) Aligned sequences of CfTX-1 (spA7L035), CfTX-2 (spA7L036), CqTX-A (spP58762), CrTX-A (spQ9GV72), and CaTX-A (spQ9GNN8) modified from Brinkman and Burnell (2007) with predicated transmembrane spanning region shown as a black line. Red letters indicated the CfTX-1 peptide synthesized by Andreosso et al (2018) that displayed chemical behaviors consistent with a transmembrane region. The blue line indicates the approximate location of motif 2 detected by MEME-suite search for these toxins (variation exists due to the spacing present in the alignment). B) Output logo from MEME-suite for motif 2 on 124 medusozoan sequences.

**Figure S2: Maximum likelihood tree for JFTs, including putative anthozoan sequences, rooted with six bacterial Cry toxins (as in Fig. 3).** Tree constructed as with Figure 3; sequences aligned using MAFFT (L-INS-I algorithm) and tree constructed using RAxML under GAMMA + I + WAG model based on the best model from ProtTest 3 with 500 rapid bootstraps replicates (all shown). JFT-1 and JFT-2 clades are labeled in black, anthozoan sequences are labeled in blue, and Cry toxins in gray.

**Figure S3: Maximum likelihood tree for JFTs including putative anthozoan sequences (rooted with sequences from the stony corals *Stylophora pistallata* and *Madracis auretenra*).** Sequences aligned and tree constructed as in Figure S2. JFT-1 subclades are labeled in black, JFT-2 subclades are labeled in gray, and anthozoan sequences are labeled in blue.

**Figure S4: Subset 1 results from aBSREL analysis for A) JFT-1 and B) JFT tests.** A) Nodes and sequences with significant test values (< 0.05) for episodic positive selection are indicated with Holm-Bonferroni corrected p-values (default for aBSREL). B) JFT-1 subset 1 gene tree and C) total JFT subset 1 gene tree are shown. Stars placed after taxa name indicate that terminal branch was predicted to be under positive selection. Stars placed at nodes found to be under significant positive selection are labeled with the corresponding node number. **‘**Exploratory’ refers to all branches being tested in the foreground. ‘JFT-1 vs. JFT-2’ indicates that JFT-1 was used as foreground and JFT-2 was used as the background; all subsequent comparative tests are in this style. Listed taxa are shown (also listed in supplimentary table S5), and tree topologies are the same as those used for BUSTED and RELAX tests. Slashes indicate no significant results for that test.

**Figure S5: Subset 2 results from aBSREL analysis for A) JFT-1 and B) JFT tests.** Similar description as Figure S4.

**Figure S6: Subset 3 results from aBSREL analysis for A) JFT-1 and B) JFT tests.** Similar description as Figure S4.
