# Supplementary figures and images for: Phylogenetic and Selection Analysis of an Expanded Family of Putatively Pore-Forming Jellyfish Toxins (Cnidaria: Medusozoa)
Source: Genome Biol Evol. 2021 Apr 23;13(6):evab081. doi: 10.1093/gbe/evab081 (PMC8214413; doi:10.1093/gbe/evab081)

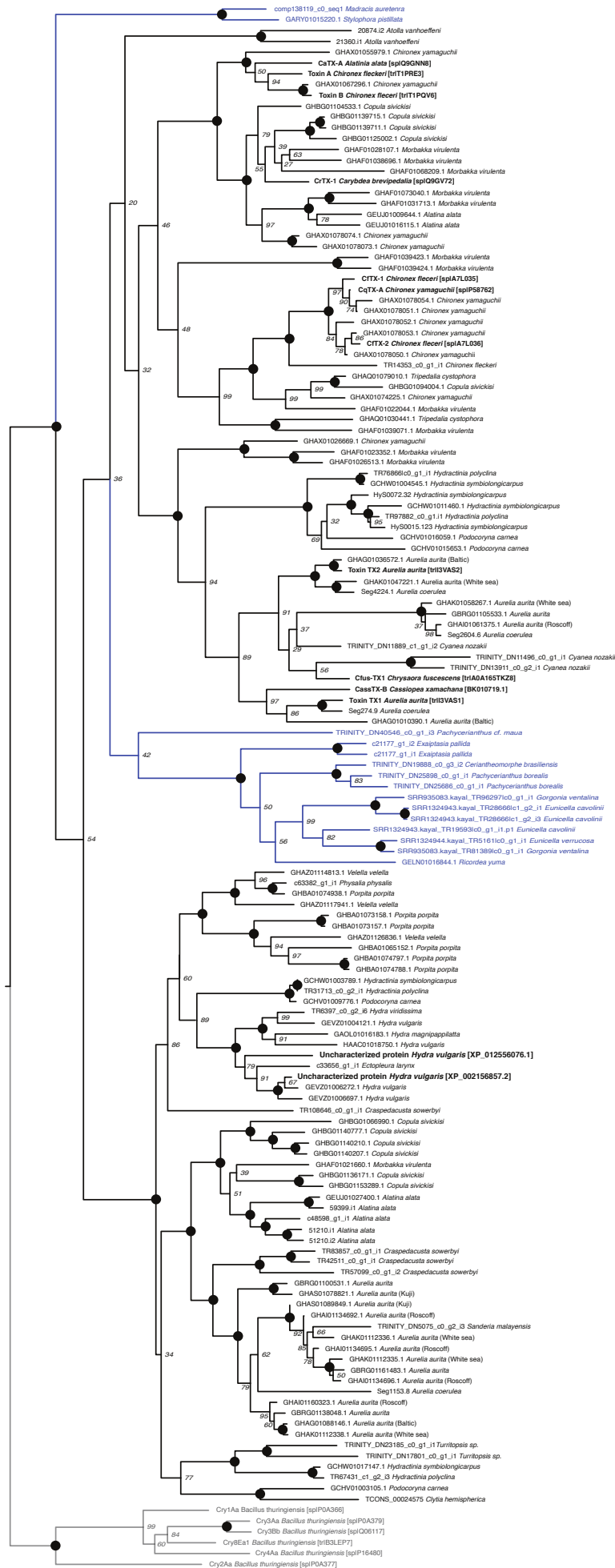

Supplement: evab081_Supplementary_Data [file evab081_supplementary_data.zip › SuppFigureS2.pdf]

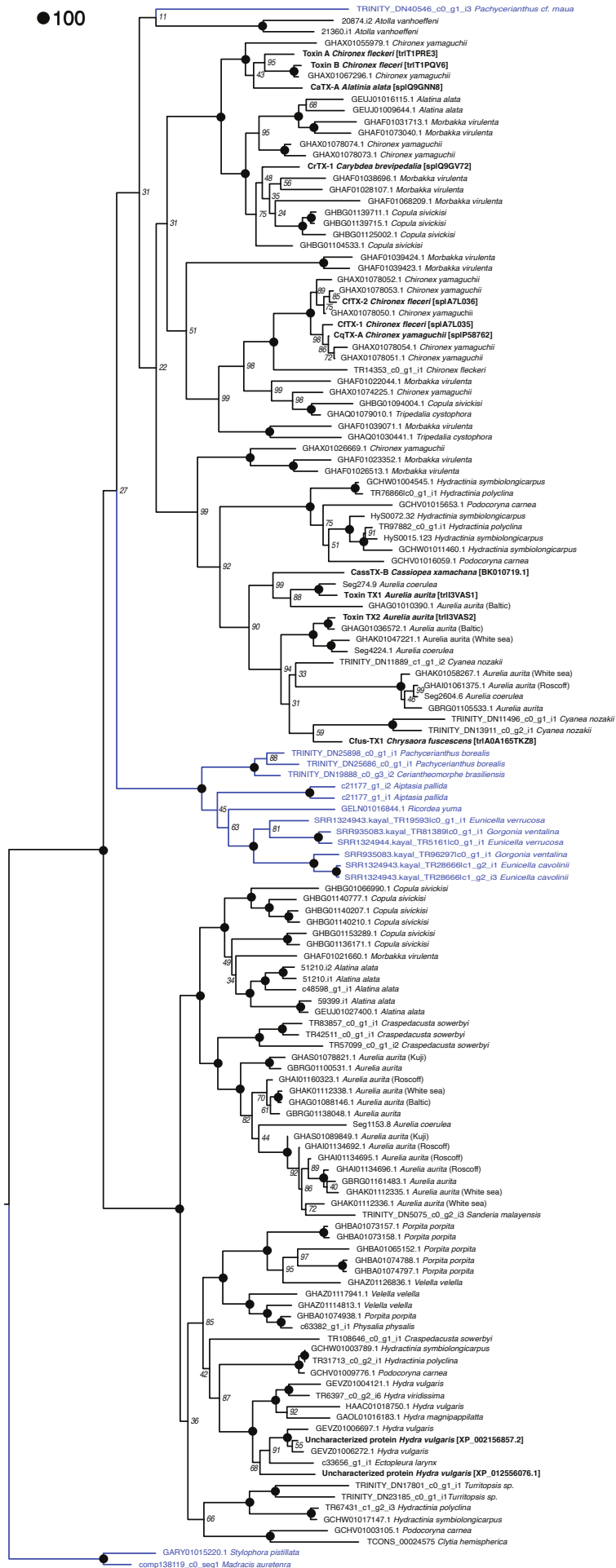

Anthozoa  
JFT-1a  
JFT-1b  
JFT-1c  
Anthozoa  
JFT-2b  
JFT-2a  
JFT-2-like  
Anthozoa

Supplement: evab081_Supplementary_Data [file evab081_supplementary_data.zip › SuppFigureS3.pdf]
